# Physical Fitness and Dyslipidemia Among Japanese: A Cohort Study From the Niigata Wellness Study

**DOI:** 10.2188/jea.JE20200034

**Published:** 2021-04-05

**Authors:** Haruki Momma, Kiminori Kato, Susumu S. Sawada, Yuko Gando, Ryoko Kawakami, Motohiko Miyachi, Ryoichi Nagatomi, Minoru Tashiro, Yasuhiro Matsubayashi, Satoru Kodama, Midori Iwanaga, Kazuya Fujihara, Hirohito Sone

**Affiliations:** 1 Department of Medicine and Science in Sports and Exercise, Tohoku University Graduate School of Medicine, Sendai, Japan; 2 Department of Hematology, Endocrinology and Metabolism, Niigata University Faculty of Medicine, Niigata, Japan; 3 Department of Physical Activity Research, National Institutes of Biomedical Innovation, Health and Nutrition, Tokyo, Japan; 4 Department of Prevention of Noncommunicable Diseases and Promotion of Health Checkup, Niigata University Graduate School of Medical and Dental Sciences, Niigata, Japan; 5 Faculty of Sport Sciences, Waseda University, Saitama, Japan; 6 Division of Biomedical Engineering for Health and Welfare, Tohoku University Graduate School of Biomedical Engineering, Sendai, Japan; 7 Niigata Association of Occupational Health, Niigata, Japan

**Keywords:** grip strength, muscle strength, balance, flexibility, reaction time, lipid

## Abstract

**Background:**

Grip strength reflects systemic muscle strength and mass and is reportedly associated with various metabolic variables. However, its prognostic association with dyslipidemia is unknown. We examined the association of grip strength and other physical fitness markers with the incidence of dyslipidemia among Japanese adults.

**Methods:**

A total of 16,149 Japanese (6,208 women) individuals aged 20–92 years who underwent a physical fitness test between April 2001 and March 2002 were included in this cohort study. Grip strength, vertical jump, single-leg balance with eyes closed, forward bending, and whole-body reaction time were evaluated at baseline. Dyslipidemia was annually determined based on fasting serum lipid profiles and self-reported dyslipidemia from April 2001 to March 2008.

**Results:**

During the follow-up period, 4,458 (44.9%) men and 2,461 (39.6%) women developed dyslipidemia. A higher relative grip strength (grip strength/body mass index) was associated with a lower incidence of dyslipidemia among both men and women (*P* for trend <0.001). Compared with those for the first septile, the hazards ratios and 95% confidence intervals (CIs) for the seventh septile were 0.56 (95% CI, 0.50–0.63) for men and 0.69 (95% CI, 0.58–0.81) for women. Moreover, relative vertical jump (vertical jump strength/body mass index) was also inversely associated with the incidence of dyslipidemia among both men and women (*P* for trend <0.001). There was no association between other physical fitness and dyslipidemia among both men and women.

**Conclusion:**

Relative grip strength and vertical jump may be useful risk markers of the incidence of dyslipidemia.

## INTRODUCTION

Dyslipidemia, defined by abnormal serum lipid levels, is a major risk factor for cardiovascular disease (CVD),^1^ the leading cause of death worldwide.^2^ Dyslipidemia is characterized by high levels of low-density lipoprotein cholesterol (LDL-C), high levels of triglycerides (TG), and/or low levels of high-density lipoprotein cholesterol (HDL-C).^3^ In addition to this lipid triad, a high level of non-HDL-C (total cholesterol [TC] minus HDL-C) is also known as a predictor of CVD mortality.^4^ Given the high prevalence, low control rate, and social burden,^5^^–^^7^ preventing dyslipidemia is a public health priority. Regular physical activity plays a favorable role in the prevention of metabolic abnormalities, and a recent guideline recommends not only aerobic but also muscle-strengthening physical activity to improve individuals’ health.^8^

Grip strength is a simple, quick, and inexpensive method to measure muscle strength in clinical practice.^9^ Recent large epidemiological studies showed that a higher grip strength was associated with better health outcomes including all-cause death, CVD death, and CVD incidence.^10^^,^^11^ Some, but not all,^12^ cross-sectional studies have reported that a lower grip strength was associated with serum lipid abnormalities.^13^^–^^19^ Although these previous studies suggested that grip strength can be a risk marker of the development of dyslipidemia, to our knowledge, no longitudinal study to date has examined the association between grip strength and the incidence of dyslipidemia. Moreover, recent studies focused on relative grip strength, not absolute strength, as a risk maker of dyslipidemia.^15^^–^^19^ Relative grip strength is generally calculated as absolute grip strength divided by body mass index (BMI),^15^^–^^19^ and has been recommended to address concomitant health risks of increased body size and low muscular strength in other fields including gerontology.^20^

This study aimed to examine the association between grip strength and the development of dyslipidemia among a Japanese population. We set the incidence of dyslipidemia and its components as the primary and secondary outcomes, respectively. In addition, physical fitness is not limited to muscle strength. Power, balance, flexibility, and reaction time are other components of physical fitness,^21^^,^^22^ and some of the measurements for these components are as simple as grip strength. Thus, we secondarily explored the association of these physical fitness with the incidence of dyslipidemia.

## METHODS

### Participants

Our study included Japanese individuals who underwent annual health examinations at the Niigata Association of Occupational Health in Niigata, Japan. The Niigata Association of Occupational Health has multiple health management centers across almost the entire area in Niigata Prefecture and has been conducting screening health examinations and routine health examinations. Basically, all individuals provide written informed consent when they attend a screening health examination or routine health examination in these centers. Details on these health examinations are provided elsewhere.^23^^,^^24^

**Figure 1.**
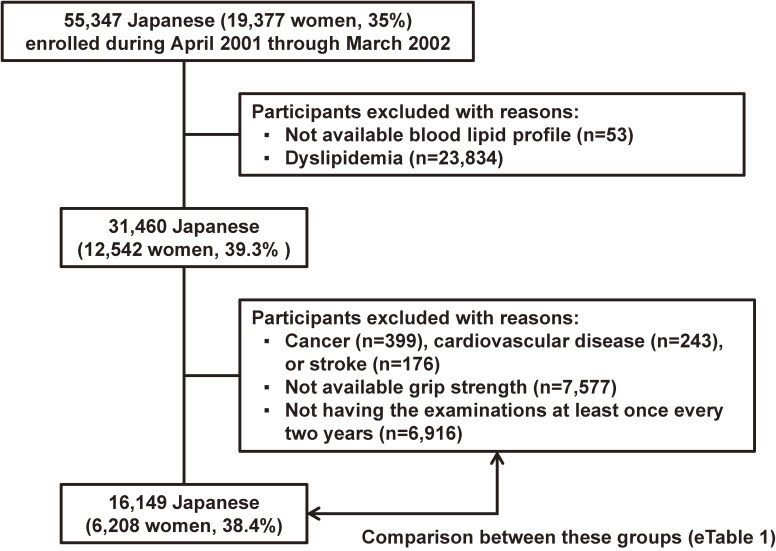
Flowchart of the selection of study participants

Our participants consisted of 55,347 Japanese individuals (19,377 women [35.0%]) who underwent an initial health examination between April 2001 and March 2002 (Figure 1). Individuals for whom no information on serum lipid profile was available (*n* = 53) or who had dyslipidemia (*n* = 23,834) at baseline were excluded. Additionally, individuals with history of cancer (*n* = 399), cardiovascular disease (*n* = 243), or stroke (*n* = 176) were excluded. We also excluded individuals who did not undergo a grip strength test at baseline (*n* = 7,577). Furthermore, individuals who did not undergo the examinations at least once every 2 years were excluded (*n* = 6,916) because 1) we were concerned about overestimating the person-period and 2) a higher examination frequency was associated with a higher incidence of dyslipidemia in our participants using a Cox proportional hazards regression analysis (*P* for trend <0.001). Finally, 16,149 individuals (6,208 women, 38.4%) aged 20–92 years were eligible for the analysis.

This study was approved by the ethics committees of the School of Medicine, Faculty of Medicine, Niigata University.

### Assessment of clinical variables

Smoking and alcohol status; exercise habit; skipping breakfast; and history of dyslipidemia, diabetes, and hypertension were assessed using a self-reported questionnaire at each annual examination. BMI (kg/m^2^) was calculated from height and weight without shoes or heavy clothing. Blood pressure was measured in a sitting position. Blood samples were collected from the median cubital vein after an overnight fast and were measured for TC, LDL-C, TG, and HDL-C using automatic clinical chemistry analyzers (HITACHI 7250, 7600, 7700; Hitachi, Tokyo, Japan) and for blood glucose and HbA1c levels (JCA-BM9030; JEOL, Tokyo, Japan). The National Glycohemoglobin Standardization Program equivalent value (%) was used to convert the HbA1c value.^25^

### Grip strength and other physical fitness tests

Physical fitness tests were conducted after a warm-up exercise at the time of the health examinations. The details of the physical fitness tests are described elsewhere.^24^

Grip strength was measured using a dynamometer (T.K.K. 5401; Takei Scientific Instruments Co., Ltd., Niigata, Japan) with the individuals in a standing position. The participants performed the test once for each hand alternately, and the highest value was used. Based on previous studies,^15^^–^^19^ we calculated the relative grip strength as absolute grip strength divided by BMI.

Lower-extremity muscle power was assessed using a vertical jump-measuring instrument (T.K.K. 5414; Takei Scientific Instruments). The jump height (cm) was assessed based on the equation of jumping time and distance. Each individual performed two trials, and the best performance was used. We used the vertical jump relative to BMI in some of the analyses to compared with the result of grip strength.

Static balance was assessed by measuring the duration (seconds) of single-leg balance with eyes closed using a stopwatch. Participants were asked to keep a standing position up to 240 seconds with their hands placed on their hips. The best value among three trials was used. A longer duration represents a better static balance.

Trunk flexibility (standing forward bending) was measured once using a digital flexibility testing device (T.K.K. 5403; Takei Scientific Instruments). Each individual was instructed to stand on a measuring bench and then push down the measurement board with both fingertips as much as possible with their legs extended. The distance (in centimeters) reached by the middle fingers was recorded. A larger positive score represents better trunk flexibility.

Whole-body reaction time was measured using a pressure-sensing mat (T.K.K. 5408; Takei Scientific Instruments). Each individual was instructed to stand on the mat and jump upright as quickly as possible in response to a red-light sign. The time (in milliseconds) between the flashing and the disappearance of foot pressure from the mat was recoded. The average of three trials was calculated. A shorter reaction time represents better whole-body reaction time.

For the analysis of physical fitness, participants were first divided into septiles (S1–S7) based on each physical fitness marker at baseline after the stratification by age (in 5-year increments) to consider the confounding effect of age.

### Dyslipidemia

Dyslipidemia was defined as hyper-LDL-cholesterolemia (≥140 mg/dL), hypertriglyceridemia (≥150 mg/dL), hypo-HDL-cholesterolemia (<40 mg/dL), hyper-non-HDL-cholesterolemia (≥170 mg/dL), or a self-reported history of previously being diagnosed with or currently taking medication for dyslipidemia.^3^ Participants were considered to have incident dyslipidemia when they met any of these conditions in the subsequent health checkups from April 2002 to March 2008.

### Statistical analysis

Multiple imputation with chained equations was used to handle missing data in this study. Analysis of imputed datasets reduces the effects of potential bias introduced by missing data.^26^^,^^27^ The percentage of missing data for physical fitness measurement at baseline ranged from 11.2% for single-leg balance to 14.0% for vertical jump. The missing values for covariates fell within 0.2% of the participants. These missing values were imputed according to a model comprising all variables used in analysis models including sensitivity analyses in addition to survival times and event status of primary and secondary outcomes.^27^^–^^29^ We created 20 multiple imputed datasets and showed pooled estimates among them.

We describe the participants’ baseline characteristics according to the septiles of relative grip strength. Here we show continuous variables as median (interquartile range) and categorical variables as number (percentage).

Cox proportional hazards regression analysis was used to examine the association between relative grip strength and the incidences of dyslipidemia and its components and calculate the hazard ratios (HRs) and 95% confidence intervals (CIs). The proportionality assumption of the models was tested using a log-minus-log plot; no evidence of violation was found. *P* values for trend were also calculated using the septiles of relative grip strength as a continuous variable. We adjusted the following as potential confounding factors at baseline: age (continuous variable), smoking (never, former, or current), alcohol (never, 1–2 days/week, 3–6 days/week, or every day), skipping breakfast (no or yes), diabetes (blood glucose ≥126 mg/dL, HbA1c ≥6.5%, or self-reported history of clinician-diagnosed diabetes [no or yes]), and hypertension (systolic blood pressure ≥140 mm Hg, diastolic blood pressure ≥90 mm Hg, or self-reported history clinician-diagnosed hypertension [no or yes]) (model 1). In addition to those in model 1, other physical fitness (continuous variables) were considered in model 2. To ensure the comparability of our study and recent previous studies,^15^^–^^19^ we did not enter a body size index in our main analyses. Instead, we performed a sensitivity analysis in which absolute grip strength was used as an exposure variable and BMI, weight, and height (continuous variable) as covariates (model 3). We conducted these analyses stratified by sex because men had a higher level of relative grip strength (*P* < 0.001) and a higher incidence of dyslipidemia (*P* < 0.001) than women. Furthermore, to explore potential effect modifications, we examined the interactions between relative grip strength and covariates by adding cross-product terms.

We also conducted several sensitivity analyses for dyslipidemia as a primary outcome. First, to eliminate the influence of possible preexisting dyslipidemia at baseline, we excluded participants who developed dyslipidemia within 2 years after the start of follow-up. Second, we used more conservative criteria for determining dyslipidemia development in which dyslipidemia was diagnosed when an individual met the criteria for dyslipidemia at least twice during the follow-up period. In this case, survival time was defined as the period until the first diagnosis was established.

For our secondary purpose, we used other physical fitness markers as explanatory variables. BMI was considered in model 3. For vertical jump, relative vertical jump was also compared with relative grip strength. We also performed a complete-case analysis to check the robustness of the results as 10% or more of the results for these fitness tests were unavailable.

Finally, we entered serum LDL-C, TG, and LDL-C level (continuous variables) into the Model 2 to examine the influence of serum lipid profile at baseline.

All of the statistical analyses were performed using SPSS version 22 (IBM Japan, Tokyo, Japan).

### Role of the funding source

This work was financially supported by the Japan Society for the Promotion of Science. The sponsor had no influence in the study design or conduct.

## RESULTS

The median (interquartile range) age of the participants at baseline was 50 (44–56) years. The median follow-up period was 3 years. During the follow-up period from April 2002 to March 2008, 4,458 (44.9%) men and 2,461 (39.6%) women developed dyslipidemia.

eTable 1 compares the characteristics of the participants included in and those excluded from the analysis. Based on the literature,^30^^,^^31^ the effect sizes of all variables were small or weak.

The baseline characteristics of the participants according to septiles of relative grip strength are shown in Table 1. The participants with S7 had favorable results of laboratory test, blood pressure, and other physical fitness compared to those with S1. The percentages of diabetes and hypertension were lower in the participants with S7.

**Table 1.** Baseline characteristics of participants according to septiles of relative grip strength

	Septiles of relative grip strength, kg/(kg/m^2^)						
	S1 (*n* = 2,300)	S2 (*n* = 2,311)	S3 (*n* = 2,311)	S4 (*n* = 2,305)	S5 (*n* = 2,312)	S6 (*n* = 2,309)	S7 (*n* = 2,301)
Men	1.51 (1.36, 1.63)	1.75 (1.61, 1.82)	1.87 (1.73, 1.95)	1.97 (1.84, 2.07)	2.09 (1.95, 2.19)	2.23 (2.08, 2.33)	2.46 (2.30, 2.60)
Women	0.94 (0.85, 1.02)	1.09 (1.02, 1.16)	1.20 (1.10, 1.26)	1.29 (1.18, 1.35)	1.37 (1.27, 1.44)	1.47 (1.36, 1.54)	1.64 (1.53, 1.74)

Sex							
Men, *n* (%)	1,416 (61.6)	1,423 (61.6)	1,422 (61.5)	1,419 (61.6)	1,423 (61.5)	1,421 (61.5)	1,417 (61.6)
Women, *n* (%)	884 (38.4)	888 (38.4)	889 (38.5)	886 (38.4)	889 (38.5)	888 (38.5)	884 (38.4)
Age, year	50.0 (44.0, 56.0)	50.0 (44.0, 57.0)	50.0 (44.0, 56.0)	50.0 (44.0, 56.0)	50.0 (44.0, 56.0)	50.0 (44.0, 56.0)	50.0 (44.0, 56.0)
Height, cm	160.1 (153.9, 166.3)	161.9 (155.4, 168.0)	162.9 (156.6, 168.7)	163.6 (156.8, 169.6)	164.1 (157.9, 169.8)	165.0 (159.0, 170.8)	166.6 (160.4, 172.7)
Weight, kg	62.5 (55.5, 69.2)	60.6 (53.7, 67.7)	59.6 (53.3, 66.4)	58.8 (52.4, 65.6)	57.4 (51.0, 64.3)	57.1 (51.2, 63.5)	55.4 (50.0, 61.8)
BMI, kg/m^2^	24.2 (22.5, 26.3)	23.3 (21.6, 24.8)	22.6 (21.1, 24.2)	22.0 (20.6, 23.6)	21.4 (19.9, 23.0)	21.0 (19.6, 22.4)	20.0 (18.7, 21.4)
SBP, mm Hg	118.0 (110.0, 130.0)	117.0 (108.0, 128.0)	116.0 (107.0, 126.0)	116.0 (107.0, 126.0)	115.0 (106.0, 125.0)	114.0 (105.0, 125.0)	114.0 (105.0, 123.0)
DBP, mm Hg	77.0 (68.0, 84.0)	76.0 (68.0, 83.0)	75.0 (68.0, 82.0)	74.0 (67.0, 82.0)	74.0 (67.0, 82.0)	74.0 (67.0, 82.0)	72.0 (67.0, 81.0)
TC, mg/dL	193.0 (176.0, 207.0)	191.0 (175.0, 206.0)	192.0 (174.0, 206.0)	190.0 (173.0, 206.5)	191.0 (172.0, 207.0)	191.0 (173.0, 207.0)	187.0 (169.0, 205.0)
TG, mg/dL	85.0 (65.0, 109.0)	81.0 (61.0, 105.0)	79.0 (60.0, 104.0)	77.0 (59.0, 101.0)	74.0 (57.0, 99.0)	74.0 (56.0, 97.0)	71.0 (54.0, 92.0)
LDL-C, mg/dL	111.0 (96.0, 124.0)	108.0 (93.0, 122.0)	108.0 (93.0, 122.0)	105.0 (89.0, 120.0)	105.0 (89.0, 120.0)	104.0 (89.0, 119.0)	101.0 (84.0, 115.0)
HDL-C, mg/dL	61.0 (52.0, 71.0)	62.0 (53.0, 72.0)	63.0 (54.0, 73.0)	63.0 (55.0, 75.0)	65.0 (56.0, 77.0)	66.0 (56.0, 77.0)	68.0 (58.0, 79.0)
Non-HDL-C, mg/dL	131.0 (115.0, 145.0)	128.0 (110.0, 143.0)	128.0 (111.0, 143.0)	125.0 (107.0, 141.0)	124.0 (107.0, 140.0)	123.0 (106.0, 139.0)	118.0 (100.0, 135.0)
Blood glucose, mg/dL	95.0 (89.0, 102.0)	94.0 (88.0, 101.0)	93.0 (88.0, 100.0)	92.0 (87.0, 99.0)	92.0 (87.0, 99.0)	92.0 (87.0, 98.0)	92.0 (87.0, 98.0)
HbA1c, %	5.2 (4.9, 5.5)	5.1 (4.9, 5.5)	5.1 (4.9, 5.5)	5.1 (4.9, 5.4)	5.1 (4.9, 5.4)	5.1 (4.9, 5.4)	5.1 (4.8, 5.4)
Smoking status, *n* (%)^a^							
Never smoker	1,234 (53.6)	1,206 (52.2)	1,202 (52.0)	1,144 (49.6)	1,132 (49.0)	1,089 (47.2)	1,099 (47.7)
Former smoker	419 (18.2)	404 (17.5)	425 (18.4)	400 (17.3)	425 (18.4)	421 (18.2)	363 (15.8)
Current smoker	647 (28.1)	701 (30.3)	684 (29.6)	761 (33.0)	755 (32.7)	799 (34.6)	839 (36.5)
Drinking status, *n* (%)^a^							
None	730 (31.8)	645 (27.9)	627 (27.1)	605 (26.2)	612 (26.5)	554 (24.0)	583 (25.3)
1–2 days/week	370 (16.1)	353 (15.3)	356 (15.4)	370 (16.1)	333 (14.4)	350 (15.2)	345 (15.0)
3–6 days/week	499 (21.7)	530 (22.9)	533 (23.1)	503 (21.8)	534 (23.1)	532 (23.1)	473 (20.6)
7 days/week	701 (30.5)	784 (33.9)	795 (34.4)	827 (35.9)	833 (36.0)	873 (37.8)	900 (39.1)
Skipping breakfast, *n* (%)							
No	2,162 (94.0)	2,182 (94.4)	2,195 (95.0)	2,189 (95.0)	2,204 (95.3)	2,174 (94.1)	2,182 (94.8)
Yes	138 (6.0)	130 (5.6)	116 (5.0)	116 (5.0)	108 (4.7)	135 (5.9)	119 (5.2)
Exercise habit, *n* (%)							
No	1,534 (66.7)	1,512 (65.4)	1,494 (64.6)	1,535 (66.6)	1,524 (65.9)	1,495 (64.8)	1,564 (68.0)
Yes	766 (33.3)	800 (34.6)	817 (35.4)	771 (33.4)	788 (34.1)	814 (35.2)	737 (32.0)
Diabetes, *n* (%)	151 (6.6)	111 (4.8)	109 (4.7)	79 (3.4)	68 (2.9)	68 (2.9)	75 (3.3)
Hypertension, *n* (%)	542 (23.6)	465 (20.1)	433 (18.7)	397 (17.2)	386 (16.7)	370 (16.0)	318 (13.8)
Grip strength, kg	32.0 (23.0, 38.0)	36.0 (26.0, 41.0)	37.0 (27.0, 43.0)	39.0 (28.0, 45.0)	40.0 (29.0, 46.0)	42.0 (31.0, 48.0)	45.0 (33.0, 51.0)
Vertical jump, cm	36.4 (30.0, 43.0)	38.0 (31.9, 45.0)	39.0 (32.5, 46.0)	39.3 (33.0, 46.0)	40.0 (34.0, 47.0)	41.0 (34.0, 47.0)	42.0 (35.9, 48.0)
Single-leg balance, sec.	28.0 (13.0, 56.9)	32.6 (14.9, 65.1)	34.2 (15.0, 67.4)	36.5 (17.0, 69.8)	40.1 (18.0, 75.6)	41.8 (19.0, 77.1)	43.0 (19.1, 82.6)
Forward bend, cm	7.0 (1.4, 12.0)	8.0 (2.0, 13.0)	8.0 (3.0, 13.2)	8.3 (3.2, 14.0)	9.0 (3.0, 14.0)	9.9 (4.0, 15.0)	10.0 (4.0, 15.0)
Reaction time, msec.	365.6 (333.5, 402.5)	358.5 (329.2, 394.8)	356.6 (326.8, 391.7)	351.0 (324.5, 387.2)	348.6 (321.4, 383.2)	347.4 (322.0, 379.9)	345.1 (317.8, 376.6)

Data are expressed as median (interquartile range) for continuous variable and number (percentage) for categorical variable.

BMI, body mass index; DBP, diastolic blood pressure; HbA1c, hemoglobin A1c; HDL-C, high-density lipoprotein cholesterol; LDL-C, low-density lipoprotein cholesterol; SBP, systolic blood pressure; TC, total cholesterol; TG, triglyceride.

^a^Numbers and percentages of participants may not sum to total numbers or 100 due to multiple imputation or rounding.

Table 2 shows the HRs and 95% CIs of the incidence of dyslipidemia among the septiles of relative grip strength among men and women. When comparing S2–S7 with S1, the HRs were lower in septiles with higher relative grip strength among both men and women (*P* for trend <0.001). This inverse association remained after the adjustment for potential confounders in Model 1 (*P* for trend <0.001) and other physical fitness in Model 2 (*P* < 0.001).

**Table 2.** Hazard ratios of the incidence of dyslipidemia according to septiles of relative grip strength among men and women

	Relative grip strength, kg/(kg/m^2^)^a^	Person-year	Case (%)	Age-adjusted HR (95% CI)	Model 1, HR (95% CI)^b^	Model 2, HR (95% CI)^c^
Men (*n* = 9,941)						
Dyslipidemia						
S1 (*n* = 1,416)	1.51 (1.36, 1.63)	3,900	720 (50.8)	1 (Reference)	1 (Reference)	1 (Reference)
S2 (*n* = 1,423)	1.75 (1.61, 1.82)	4,137	682 (47.9)	0.90 (0.82, 1.00)	0.91 (0.82, 1.01)	0.90 (0.81, 0.998)
S3 (*n* = 1,422)	1.87 (1.73, 1.95)	4,253	675 (47.5)	0.87 (0.78, 0.97)	0.88 (0.79, 0.98)	0.87 (0.78, 0.96)
S4 (*n* = 1,419)	1.97 (1.84, 2.07)	4,383	657 (46.3)	0.83 (0.74, 0.92)	0.83 (0.75, 0.93)	0.82 (0.73, 0.91)
S5 (*n* = 1,423)	2.09 (1.95, 2.19)	4,556	619 (43.5)	0.76 (0.68, 0.84)	0.77 (0.69, 0.85)	0.75 (0.67, 0.83)
S6 (*n* = 1,421)	2.23 (2.08, 2.33)	4,627	597 (42.0)	0.72 (0.65, 0.80)	0.73 (0.65, 0.81)	0.71 (0.63, 0.79)
S7 (*n* = 1,417)	2.46 (2.30, 2.60)	4,929	508 (35.9)	0.58 (0.52, 0.65)	0.58 (0.52, 0.65)	0.56 (0.50, 0.63)
*P* for trend				<0.001	<0.001	<0.001

Women (*n* = 6,208)						
Dyslipidemia						
S1 (*n* = 884)	0.94 (0.85, 1.02)	2,839	393 (44.5)	1 (Reference)	1 (Reference)	1 (Reference)
S2 (*n* = 888)	1.09 (1.02, 1.16)	2,985	374 (42.1)	0.92 (0.80, 1.06)	0.94 (0.82, 1.08)	0.95 (0.82, 1.09)
S3 (*n* = 889)	1.20 (1.10, 1.26)	2,978	356 (40.0)	0.87 (0.76, 1.01)	0.90 (0.78, 1.03)	0.90 (0.78, 1.04)
S4 (*n* = 886)	1.29 (1.18, 1.35)	3,076	357 (40.3)	0.85 (0.74, 0.98)	0.87 (0.76, 1.01)	0.88 (0.75, 1.01)
S5 (*n* = 889)	1.37 (1.27, 1.44)	3,025	357 (40.2)	0.88 (0.77, 1.02)	0.90 (0.78, 1.04)	0.91 (0.78, 1.05)
S6 (*n* = 888)	1.47 (1.36, 1.54)	3,118	345 (38.9)	0.83 (0.72, 0.96)	0.86 (0.75, 0.998)	0.87 (0.74, 1.01)
S7 (*n* = 884)	1.64 (1.53, 1.74)	3,158	279 (31.6)	0.65 (0.56, 0.76)	0.68 (0.58, 0.79)	0.69 (0.58, 0.81)
*P* for trend				<0.001	<0.001	<0.001

CI, confidence interval; HR, hazard ratio.

^a^Data are expressed as median (interquartile range).

^b^Adjusted for age (continuous variable), smoking status (never smoker, former smoker, or current smoker), drinking status (none, 1–3 days/week, 4–6 days/week, or 7 days/week), breakfast skipping (no or yes), diabetes (no or yes), and hypertension (no or yes).

^c^Additionally adjusted for vertical jump (continuous variable), single-leg balance (continuous variable), forward bend (continuous variable), and reaction time (continuous variable).

Here, we confirmed the interaction between relative grip strength and age (*P* < 0.001). Therefore, a subgroup analysis by age (<50 and ≥50 years) was performed (eTable 2) because the median ages of the men and women were 51 and 48 years, respectively. For men in the <50 and ≥50 years groups, an inverse association between relative grip strength and dyslipidemia was consistently obtained, while among women aged ≥50 years, this association was not confirmed. No other interaction between relative grip strength and covariates was found.

The sensitivity analysis excluding participants who developed dyslipidemia within 2 years after the start of follow-up showed an inverse association between relative grip strength and the incidence of dyslipidemia (eTable 3). In addition, even when more conservative criteria for dyslipidemia were used, we obtained a similar result (eTable 4). For absolute grip strength (eTable 5), we also confirmed inverse associations in men when body weight or BMI was considered.

For the component of dyslipidemia as a secondary outcome, a higher relative grip strength was associated with a lower incidence of hyper-LDL-cholesterolemia, hypertriglyceridemia, hypo-HDL-cholesterolemia, and hyper-non-HDL-cholesterolemia in men (*P* < 0.001, Table 3) and women (*P* < 0.028, Table 4).

**Table 3.** Hazard ratios of the incidence of dyslipidemia’s components according to septiles of relative grip strength among men

	Relative grip strength, kg/(kg/m^2^)^a^	Person-year	Case (%)	Age-adjusted HR (95% CI)	Model 1, HR (95% CI)^b^	Model 2, HR (95% CI)^c^
Hyper LDL cholesterolemia						
S1 (*n* = 1,416)	1.51 (1.36, 1.63)	4,238	446 (31.5)	1 (Reference)	1 (Reference)	1 (Reference)
S2 (*n* = 1,423)	1.75 (1.61, 1.82)	4,410	399 (28.0)	0.86 (0.76, 0.99)	0.87 (0.76, 0.99)	0.85 (0.74, 0.98)
S3 (*n* = 1,422)	1.87 (1.73, 1.95)	4,591	415 (29.2)	0.86 (0.75, 0.98)	0.87 (0.76, 0.997)	0.85 (0.75, 0.98)
S4 (*n* = 1,419)	1.97 (1.84, 2.07)	4,700	363 (25.6)	0.73 (0.64, 0.84)	0.75 (0.65, 0.86)	0.73 (0.63, 0.84)
S5 (*n* = 1,423)	2.09 (1.95, 2.19)	4,799	378 (26.6)	0.75 (0.66, 0.86)	0.77 (0.67, 0.89)	0.75 (0.65, 0.86)
S6 (*n* = 1,421)	2.23 (2.08, 2.33)	4,885	358 (25.2)	0.70 (0.61, 0.80)	0.72 (0.63, 0.83)	0.70 (0.60, 0.80)
S7 (*n* = 1,417)	2.46 (2.30, 2.60)	5,117	305 (21.5)	0.57 (0.49, 0.66)	0.58 (0.50, 0.67)	0.55 (0.48, 0.64)
*P* for trend				<0.001	0.001	<0.001

Hypertriglyceridemia						
S1 (*n* = 1,416)	1.51 (1.36, 1.63)	4,226	412 (29.1)	1 (Reference)	1 (Reference)	1 (Reference)
S2 (*n* = 1,423)	1.75 (1.61, 1.82)	4,419	396 (27.8)	0.93 (0.81, 1.06)	0.93 (0.81, 1.07)	0.92 (0.80, 1.06)
S3 (*n* = 1,422)	1.87 (1.73, 1.95)	4,560	390 (27.4)	0.88 (0.77, 1.01)	0.89 (0.77, 1.02)	0.87 (0.76, 1.00)
S4 (*n* = 1,419)	1.97 (1.84, 2.07)	4,630	393 (27.7)	0.88 (0.76, 1.01)	0.86 (0.75, 0.99)	0.85 (0.74, 0.98)
S5 (*n* = 1,423)	2.09 (1.95, 2.19)	4,804	356 (25.0)	0.77 (0.67, 0.89)	0.77 (0.66, 0.88)	0.75 (0.65, 0.87)
S6 (*n* = 1,421)	2.23 (2.08, 2.33)	4,836	337 (23.7)	0.72 (0.63, 0.83)	0.71 (0.61, 0.82)	0.70 (0.60, 0.81)
S7 (*n* = 1,417)	2.46 (2.30, 2.60)	5,112	270 (19.1)	0.55 (0.47, 0.64)	0.54 (0.46, 0.63)	0.52 (0.44, 0.61)
*P* for trend				<0.001	<0.001	<0.001

Hypo HDL cholesterolemia						
S1 (*n* = 1,416)	1.51 (1.36, 1.63)	4,322	132 (9.3)	1 (Reference)	1 (Reference)	1 (Reference)
S2 (*n* = 1,423)	1.75 (1.61, 1.82)	4,523	129 (9.1)	0.94 (0.74, 1.20)	0.96 (0.76, 1.23)	0.95 (0.74, 1.21)
S3 (*n* = 1,422)	1.87 (1.73, 1.95)	4,645	127 (8.9)	0.91 (0.71, 1.15)	0.96 (0.75, 1.22)	0.94 (0.73, 1.20)
S4 (*n* = 1,419)	1.97 (1.84, 2.07)	4,745	105 (7.4)	0.74 (0.57, 0.95)	0.77 (0.59, 0.99)	0.75 (0.58, 0.98)
S5 (*n* = 1,423)	2.09 (1.95, 2.19)	4,908	99 (7.0)	0.68 (0.52, 0.88)	0.72 (0.55, 0.93)	0.70 (0.53, 0.91)
S6 (*n* = 1,421)	2.23 (2.08, 2.33)	4,969	72 (5.1)	0.49 (0.37, 0.65)	0.51 (0.38, 0.68)	0.50 (0.37, 0.67)
S7 (*n* = 1,417)	2.46 (2.30, 2.60)	5,186	62 (4.4)	0.40 (0.30, 0.55)	0.42 (0.31, 0.56)	0.40 (0.29, 0.54)
*P* for trend				<0.001	<0.001	<0.001

Hyper non-HDL cholesterolemia						
S1 (*n* = 1,416)	1.51 (1.36, 1.63)	4,333	298 (21.0)	1 (Reference)	1 (Reference)	1 (Reference)
S2 (*n* = 1,423)	1.75 (1.61, 1.82)	4,509	244 (17.1)	0.79 (0.67, 0.94)	0.80 (0.67, 0.94)	0.80 (0.67, 0.94)
S3 (*n* = 1,422)	1.87 (1.73, 1.95)	4,636	246 (17.3)	0.78 (0.66, 0.92)	0.79 (0.67, 0.94)	0.79 (0.67, 0.94)
S4 (*n* = 1,419)	1.97 (1.84, 2.07)	4,815	239 (16.8)	0.73 (0.62, 0.87)	0.75 (0.63, 0.89)	0.75 (0.63, 0.89)
S5 (*n* = 1,423)	2.09 (1.95, 2.19)	4,932	234 (16.4)	0.70 (0.59, 0.84)	0.72 (0.61, 0.85)	0.72 (0.60, 0.86)
S6 (*n* = 1,421)	2.23 (2.08, 2.33)	5,001	227 (16.0)	0.67 (0.57, 0.80)	0.69 (0.58, 0.82)	0.69 (0.58, 0.83)
S7 (*n* = 1,417)	2.46 (2.30, 2.60)	5,186	179 (12.6)	0.51 (0.43, 0.62)	0.52 (0.43, 0.63)	0.52 (0.43, 0.63)
*P* for trend				<0.001	<0.001	<0.001

CI, confidence interval; HR, hazard ratio.

^a^Data are expressed as median (interquartile range).

^b^Adjusted for age (continuous variable), smoking status (never smoker, former smoker, or current smoker), drinking status (none, 1–3 days/week, 4–6 days/week, or 7 days/week), breakfast skipping (no or yes), diabetes (no or yes), and hypertension (no or yes).

^c^Additionally adjusted for vertical jump (continuous variable), single-leg balance (continuous variable), forward bend (continuous variable), and reaction time (continuous variable).

**Table 4.** Hazard ratios of the incidence of dyslipidemia’s components according to septiles of relative grip strength among women

	Relative grip strength, kg/(kg/m^2^)^a^	Person-year	Case (%)	Age-adjusted HR (95% CI)	Model 1, HR (95% CI)^b^	Model 2, HR (95% CI)^c^
Hyper LDL cholesterolemia						
S1 (*n* = 884)	0.94 (0.85, 1.02)	2,907	341 (38.6)	1 (Reference)	1 (Reference)	1 (Reference)
S2 (*n* = 888)	1.09 (1.02, 1.16)	3,063	318 (35.8)	0.90 (0.77, 1.05)	0.92 (0.79, 1.07)	0.93 (0.79, 1.08)
S3 (*n* = 889)	1.20 (1.10, 1.26)	3,069	313 (35.2)	0.88 (0.75, 1.02)	0.90 (0.77, 1.05)	0.91 (0.78, 1.06)
S4 (*n* = 886)	1.29 (1.18, 1.35)	3,131	311 (35.1)	0.86 (0.74, 1.00)	0.88 (0.76, 1.03)	0.89 (0.76, 1.04)
S5 (*n* = 889)	1.37 (1.27, 1.44)	3,102	313 (35.2)	0.89 (0.76, 1.04)	0.91 (0.78, 1.07)	0.92 (0.79, 1.08)
S6 (*n* = 888)	1.47 (1.36, 1.54)	3,162	306 (34.5)	0.85 (0.72, 0.99)	0.89 (0.76, 1.04)	0.90 (0.77, 1.06)
S7 (*n* = 884)	1.64 (1.53, 1.74)	3,184	251 (28.4)	0.68 (0.58, 0.81)	0.72 (0.61, 0.84)	0.73 (0.61, 0.87)
*P* for trend				<0.001	0.001	0.004

Hypertriglyceridemia						
S1 (*n* = 884)	0.94 (0.85, 1.02)	3,100	122 (13.8)	1 (Reference)	1 (Reference)	1 (Reference)
S2 (*n* = 888)	1.09 (1.02, 1.16)	3,168	102 (11.5)	0.83 (0.64, 1.07)	0.83 (0.63, 1.08)	0.83 (0.64, 1.08)
S3 (*n* = 889)	1.20 (1.10, 1.26)	3,217	102 (11.5)	0.80 (0.62, 1.05)	0.82 (0.63, 1.07)	0.81 (0.62, 1.07)
S4 (*n* = 886)	1.29 (1.18, 1.35)	3,237	75 (8.5)	0.59 (0.44, 0.79)	0.59 (0.44, 0.79)	0.59 (0.44, 0.79)
S5 (*n* = 889)	1.37 (1.27, 1.44)	3,212	69 (7.8)	0.56 (0.42, 0.75)	0.56 (0.41, 0.75)	0.55 (0.41, 0.75)
S6 (*n* = 888)	1.47 (1.36, 1.54)	3,272	79 (8.9)	0.62 (0.47, 0.83)	0.64 (0.48, 0.85)	0.63 (0.47, 0.85)
S7 (*n* = 884)	1.64 (1.53, 1.74)	3,301	51 (5.8)	0.39 (0.28, 0.55)	0.40 (0.29, 0.56)	0.40 (0.28, 0.56)
*P* for trend				<0.001	<0.001	<0.001

Hypo HDL cholesterolemia						
S1 (*n* = 884)	0.94 (0.85, 1.02)	3,132	24 (2.7)	1 (Reference)	1 (Reference)	1 (Reference)
S2 (*n* = 888)	1.09 (1.02, 1.16)	3,207	20 (2.3)	0.82 (0.45, 1.48)	0.87 (0.48, 1.58)	0.93 (0.51, 1.70)
S3 (*n* = 889)	1.20 (1.10, 1.26)	3,224	19 (2.1)	0.77 (0.42, 1.41)	0.84 (0.46, 1.54)	0.92 (0.50, 1.70)
S4 (*n* = 886)	1.29 (1.18, 1.35)	3,269	7 (0.8)	0.28 (0.12, 0.66)	0.31 (0.13, 0.73)	0.35 (0.15, 0.82)
S5 (*n* = 889)	1.37 (1.27, 1.44)	3,235	14 (1.6)	0.58 (0.30, 1.11)	0.62 (0.32, 1.21)	0.71 (0.36, 1.42)
S6 (*n* = 888)	1.47 (1.36, 1.54)	3,290	10 (1.1)	0.40 (0.19, 0.85)	0.46 (0.22, 0.96)	0.53 (0.25, 1.15)
S7 (*n* = 884)	1.64 (1.53, 1.74)	3,303	10 (1.1)	0.40 (0.19, 0.84)	0.45 (0.22, 0.95)	0.55 (0.25, 1.21)
*P* for trend				0.001	0.003	0.028

Hyper non-HDL cholesterolemia						
S1 (*n* = 884)	0.94 (0.85, 1.02)	3,077	208 (23.5)	1 (Reference)	1 (Reference)	1 (Reference)
S2 (*n* = 888)	1.09 (1.02, 1.16)	3,171	179 (20.2)	0.85 (0.69, 1.03)	0.86 (0.71, 1.06)	0.88 (0.72, 1.08)
S3 (*n* = 889)	1.20 (1.10, 1.26)	3,203	189 (21.3)	0.88 (0.72, 1.07)	0.91 (0.74, 1.10)	0.92 (0.76, 1.13)
S4 (*n* = 886)	1.29 (1.18, 1.35)	3,303	185 (20.9)	0.85 (0.70, 1.03)	0.88 (0.72, 1.07)	0.90 (0.73, 1.10)
S5 (*n* = 889)	1.37 (1.27, 1.44)	3,256	181 (20.4)	0.85 (0.70, 1.04)	0.87 (0.71, 1.06)	0.90 (0.73, 1.10)
S6 (*n* = 888)	1.47 (1.36, 1.54)	3,300	162 (18.2)	0.75 (0.61, 0.92)	0.79 (0.64, 0.97)	0.82 (0.66, 1.01)
S7 (*n* = 884)	1.64 (1.53, 1.74)	3,310	150 (17.0)	0.69 (0.56, 0.85)	0.72 (0.58, 0.89)	0.76 (0.60, 0.95)
*P* for trend				<0.001	0.003	0.021

CI, confidence interval; HR, hazard ratio.

^a^Data are expressed as median (interquartile range).

^b^Adjusted for age (continuous variable), smoking status (never smoker, former smoker, or current smoker), drinking status (none, 1–3 days/week, 4–6 days/week, or 7 days/week), breakfast skipping (no or yes), diabetes (no or yes), and hypertension (no or yes).

^c^Additionally adjusted for vertical jump (continuous variable), single-leg balance (continuous variable), forward bend (continuous variable), and reaction time (continuous variable).

eTable 6 shows the HRs and 95% CIs of the incidence of dyslipidemia among the septiles of other physical fitness markers. Among men, better trunk flexibility was associated with a lower incidence of dyslipidemia, although this association disappeared when the complete-case analysis was conducted (*P* = 0.081, eTable 7). There was no clear association between absolute vertical jump and the incidence of dyslipidemia. However, when using a relative value to BMI, vertical jump clearly showed an inverse associated with dyslipidemia in both men (*P* for trend <0.001) and women (*P* for trend <0.001) (Table 5). Similar results were found when the complete-case analysis was performed (eTable 7).

**Table 5.** Hazard ratios of the incidence of dyslipidemia according to septiles of relative vertical jump among men and women

	Relative vertical jump, cm/(kg/m^2^)^a^	Person-year	Case (%)	Age-adjusted HR (95% CI)	Model 1, HR (95% CI)^b^	Model 2, HR (95% CI)^c^
Men (*n* = 9,941)						
Dyslipidemia						
S1 (*n* = 1,419)	1.50 (1.28, 1.64)	3,990	691 (48.7)	1 (Reference)	1 (Reference)	1 (Reference)
S2 (*n* = 1,421)	1.73 (1.60, 1.89)	4,096	716 (50.4)	1.02 (0.91, 1.14)	1.03 (0.92, 1.16)	1.03 (0.92, 1.15)
S3 (*n* = 1,421)	1.86 (1.73, 2.03)	4,292	693 (48.7)	0.94 (0.84, 1.06)	0.96 (0.85, 1.07)	0.95 (0.85, 1.07)
S4 (*n* = 1,420)	1.97 (1.84, 2.18)	4,351	647 (45.5)	0.87 (0.78, 0.98)	0.89 (0.79, 0.99)	0.89 (0.79, 0.996)
S5 (*n* = 1,422)	2.09 (1.96, 2.31)	4,573	614 (43.2)	0.79 (0.70, 0.89)	0.81 (0.71, 0.91)	0.81 (0.71, 0.91)
S6 (*n* = 1,422)	2.25 (2.10, 2.48)	4,569	607 (42.7)	0.78 (0.69, 0.88)	0.79 (0.70, 0.89)	0.79 (0.70, 0.89)
S7 (*n* = 1,417)	2.56 (2.34, 2.84)	4,915	491 (34.7)	0.60 (0.53, 0.67)	0.60 (0.53, 0.68)	0.60 (0.53, 0.68)
*P* for trend				<0.001	<0.001	<0.001

Women (*n* = 6,208)						
Dyslipidemia						
S1 (*n* = 885)	1.06 (0.92, 1.20)	2,756	422 (47.7)	1 (Reference)	1 (Reference)	1 (Reference)
S2 (*n* = 888)	1.28 (1.16, 1.41)	2,978	380 (42.8)	0.83 (0.72, 0.97)	0.85 (0.73, 0.997)	0.84 (0.72, 0.98)
S3 (*n* = 888)	1.41 (1.29, 1.55)	2,910	366 (41.2)	0.83 (0.71, 0.96)	0.85 (0.73, 0.99)	0.84 (0.72, 0.98)
S4 (*n* = 886)	1.53 (1.39, 1.67)	3,022	375 (42.3)	0.82 (0.70, 0.95)	0.85 (0.73, 0.99)	0.83 (0.71, 0.97)
S5 (*n* = 889)	1.63 (1.50, 1.78)	3,107	314 (35.4)	0.67 (0.57, 0.79)	0.70 (0.59, 0.82)	0.68 (0.58, 0.80)
S6 (*n* = 888)	1.77 (1.63, 1.91)	3,204	322 (36.3)	0.68 (0.58, 0.80)	0.70 (0.60, 0.83)	0.68 (0.58, 0.81)
S7 (*n* = 885)	2.02 (1.85, 2.21)	3,205	282 (31.9)	0.59 (0.50, 0.70)	0.62 (0.52, 0.73)	0.60 (0.50, 0.71)
*P* for trend				<0.001	<0.001	<0.001

CI, confidence interval; HR, hazard ratio.

The numbers of participants and cases may not sum to the numbers shown in the text due to multiple imputation.

^a^Data are expressed as median (interquartile range).

^b^Adjusted for age (continuous variable), smoking status (never smoker, former smoker, or current smoker), drinking status (none, 1–3 days/week, 4–6 days/week, or 7 days/week), breakfast skipping (no or yes), diabetes (no or yes), and hypertension (no or yes).

^c^Additionally adjusted for grip strength (continuous variable), single-leg balance (continuous variable), forward bend (continuous variable), and reaction time (continuous variable).

Finally, the association of relative grip strength and vertical jump with the incidence of dyslipidemia was considerably attenuated when adjusted for serum lipid levels at baseline, particularly in women. However, men in the highest septile still had a lower incidence of dyslipidemia compared with men in the lowest septile (eTable 8).

## DISCUSSION

Here, we conducted a cohort study to examine the association between grip strength and the development of dyslipidemia among Japanese adults. There was an inverse dose-response association between relative grip strength and the incidence of dyslipidemia. Moreover, a higher relative grip strength was associated with a lower incidence of hyper-LDL-cholesterolemia, hypertriglyceridemia, hypo-HDL-cholesterolemia, and hyper-non-HDL-cholesterolemia. We also explored the association between other physical fitness and the incidence of dyslipidemia as a secondary purpose. Relative, but not absolute, vertical jump was inversely associated with the incidence of dyslipidemia.

Grip strength reflects overall muscle strength and mass. Muscle strength can be improved by muscle-strengthening activities, and resistance training, a typical muscle-strengthening activity, promotes decreases in TC, TG, and LDL-C levels and increases in HDL-C level.^32^ In addition, skeletal muscle is a main tissue of fatty acid uptake and oxidation.^33^ Taken together, grip strength may be a risk marker of the incidence of dyslipidemia and its components. Several previous cross-sectional studies showed that a lower grip strength was associated with serum lipid abnormalities.^13^^–^^19^ For example, a higher relative grip strength was associated with a lower TG level and a higher HDL-C level in Americans.^15^ Similar findings were obtained in Asians.^16^^,^^17^ Moreover, a recent study reported that relative grip strength was inversely associated with the prevalence of dyslipidemia and hyperlipidemia defined by Chinese guidelines.^18^ Consistent with previous findings, our study showed that relative grip strength was inversely associated with dyslipidemia and its components. In contrast to the above results, Aoyama et al reported that absolute grip strength was not associated with TG and HDL-C among Japanese adults.^12^ This discrepancy may be explained by the use of absolute versus relative grip strength. Some health outcomes may be more strongly associated with relative measures defined by muscle strength and body mass (relative strength) or muscle mass (muscle quality).^20^^,^^34^ In addition, Li et al showed that a higher absolute handgrip strength was associated with a higher, not lower, prevalence of dyslipidemia and hyperlipidemia.^18^ This association was also confirmed in our sensitivity analysis (eTable 5). These paradoxical associations potentially resulted from confounders related to body size. Indeed, absolute grip strength was positively associated with BMI in this study (*r* = 0.20). Taken together, these findings suggest that body size may influence the association between grip strength and dyslipidemia and that relative grip strength, but not absolute strength, can be a risk marker of the development of dyslipidemia and its components in clinical settings.

Although there was an inverse association between relative grip strength and the incidence of dyslipidemia among men regardless of age, this association was substantially attenuated and was not confirmed among women aged ≥50 years. One of the explanations for this observation may be a misclassification of participants according to relative grip strength, because the group of women aged ≥50 years had the narrowest range of relative grip strength (1.74 kg/[kg/m^2^]) among all groups (2.02–2.59 kg/[kg/m^2^]). In particular, a failure to grade participants on relative grip strength may occur among the intermediate groups (S2–S6). Another explanation may be the influence of menopause on blood lipid profile. A previous study showed that a higher prevalence of dyslipidemia was observed in postmenopause than in premenopause.^35^ Moreover, serum LDL-C and TG levels increased in women aged 50–70 years, and the lipid levels decreased after the peak age,^36^ while grip strength peaked in women aged approximately 30 years and then constantly decreased.^37^ Considering that the loss of muscle mass and strength appears to be concurrent with the occurrence of menopause,^38^ there is a possibility that lower septiles of relative grip strength among women aged ≥50 years might include more posmenopausal women compared with higher septiles.

Because muscle power has many determinants in common with muscle strength, it is easy to assume that vertical jump also associated with the risk of dyslipidemia. As expected, a higher relative vertical jump was associated with a lower incidence of dyslipidemia; this association was as strong as relative grip strength. Therefore, like grip strength, vertical jump may reflect the level of muscle-strengthening activities among our participants.

When serum lipid concentrations were adjusted for, the inverse associations of relative grip strength and vertical jump with the incidence of dyslipidemia were considerably attenuated. These findings were expected because the level of relative grip strength had already been associated with the baseline concentrations of serum lipids in this study, consistent with previous studies.^13^^–^^19^ Nevertheless, higher relative grip strength and vertical jump were associated with lower risks of dyslipidemia in men. Statistically adjusting serum lipid levels at baseline means holding serum lipids level at baseline constant among the septile groups in the analysis. In other words, it is possible to assume that the baseline serum lipid concentrations are comparable among the septile groups after adjustment for baseline serum lipid concentrations. Based on this assumption, one of the reasons why the incidence of dyslipidemia varied across the septile groups may be that the increase rates of serum lipids during the follow-up period (namely with aging) in fit men were lower than those of unfit men. Additionally, the hazard ratios in this case may represent a net influence of fitness level on the increase rates of serum lipids during the follow-up period. Because the follow-up period was short (the median and maximal follow-up period, 3 and 6 years, respectively), the influence of fitness level on the increase rates of serum lipids during the follow-up period may not be large. Further studies with longer follow-up periods are required to examine this point.

In this study, flexibility was also associated with the risk of dyslipidemia. However, it should be carefully judged whether these associations are true because this association: (1) disappeared when the complete-case analysis was conducted; and (2) was not stronger than those of relative grip strength and vertical jump. In addition, multiple testing may have led to more false positives due to type 1 error. Given these concerns, further studies are needed to confirm our findings.

This study has several limitations. First, detailed information of dyslipidemia drugs was unavailable. Therefore, we could not discriminate among participants taking lipid-lowering medication. However, we consistently confirmed inverse associations between relative grip strength and the incidence of lipid abnormalities defined by serum lipid profile only. Moreover, a total of only 358 (5.2%) participants reported their self-reported history or current medication for dyslipidemia without an abnormal lipid profile between April 2002 and March 2008. Therefore, self-reported history of dyslipidemia and current treatment of lipid-lowering drugs in this study may be not relatively accurate. Considering this, the possibility of misclassification of participants who did not report a current treatment of lipid-lowering drugs despite taking medications becomes an issue, leading to an underestimation of dyslipidemia development and a failure to exclude participants with dyslipidemia at baseline. Second, although we considered several potential confounding factors, we did not rule out the influence of unmeasured confounding factors such as dietary habits in addition to menopausal status. It is possible that highly fit individuals have healthier dietary intakes than unfit individuals. Third, because the participants in this study all attended an initial health examination between April 2001 and March 2002 and were not individuals randomly selected from the population, they may not be representative of the general population. Furthermore, only 29.2% of all participants who underwent the health examination at baseline were included in the analyses. This exclusion could have resulted in substantial selection bias, although the difference in baseline characteristics between the included and excluded participants was not large. Fourth, the present study included Japanese individuals who attended a screening health examination or routine health examination. This might lead to a selection bias because such individuals might pay more attention to their health or lifestyle compared with those who do not undergo health examinations. Thus, the generalizability of our findings might be further reduced and limited to health-conscious participants. However, routine health examinations are generally common in Japan. Moreover, considering that our results are consistent with those of previous cross-sectional studies examining the association between relative grip strength and the incidence of dyslipidemia in other races, our finding is likely applicable to other populations including other regions and races. Finally, this was an observational study. Therefore, our findings did not directly provide evidence that improving muscle strength and power can lead to a reduction in the risk of dyslipidemia.

In conclusion, relative grip strength was inversely associated with the risk of dyslipidemia and its components among Japanese adults. In addition, relative vertical jump was associated with the risk of dyslipidemia. Our results must be replicated in further studies with longer follow-up periods in the general population and in other races.

## APPENDIX A. SUPPLEMENTARY DATA

eTable 1. Difference of baseline characteristics among the included and excluded participants

eTable 2. Hazard ratios of the incidence of dyslipidemia according to septiles of relative grip strength in a sub-group analysis by age among men and women

eTable 3. Hazard ratios of the incidence of dyslipidemia according to septiles of relative grip strength excluding those who developed dyslipidemia within 2-year after the onset of follow-up among men and women

eTable 4. Hazard ratios of the incidence of dyslipidemia according to septiles of relative grip strength using a more conservative criteria for dyslipidemia among men and women

eTable 5. Hazard ratios of the incidence of dyslipidemia according to septiles of absolute grip strength among men and women

eTable 6. Association between the septiles of each physical fitness and the incidence of dyslipidemia among men and women

eTable 7. Hazard ratios of the incidence of dyslipidemia according to septiles of relative vertical jump and trunk flexibility in complete-cases analysis

eTable 8. Hazard ratios of the incidence of dyslipidemia according to septiles of relative grip strength and vertical jump after adjusted for serum lipid profile at baseline
