# Supplementary material for: Physical Fitness and Dyslipidemia Among Japanese: A Cohort Study From the Niigata Wellness Study
Source: J Epidemiol. 2021 Apr 5;31(4):287–96. doi: 10.2188/jea.JE20200034 (PMC7940973; doi:10.2188/jea.JE20200034)
Supplement: Supplementary file 1 [file je-31-287-s001.pdf]

eTable 1. Difference of baseline characteristics among the included and excluded participants

	Excluded (n= 15,311)	Included (n= 16,149)	Effect size[a]
Women, n (%)	13,169 (33.6)	6,208 (38.4)	0.03
Age, year	51.0 (44.0, 58.0)	50.0 (44.0, 56.0)	-0.12
Height, cm	162.5 (156.2, 168.7)	163.5 (157.0, 169.6)	0.10
Weight, kg	57.8 (51.5, 65.0)	58.7 (58.7, 65.6)	0.07
BMI, kg/m^2	21.9 (20.1, 23.9)	22.0 (20.3, 23.9)	0.001
SBP, mmHg	116.0 (107.0, 127.0)	116.0 (107.0, 126.0)	-0.06
Missing data	0 (0)	1 (0.006)	
DBP, mmHg	75.0 (67.0, 82.0)	75.0 (67.3, 82.0)	-0.02
Missing data	0 (0)	1 (0.006)	
TC, mg/dL	190.0 (173.0, 207.0)	191.0 (173.0, 207.0)	-0.004
Missing data	0 (0)	0 (0)	
TG mg/dL	76.0 (58.0, 100.0)	77.0 (59.0, 101.0)	0.02
Missing data	0 (0)	0 (0)	
LDL-C, mg/dL	106.0 (90.0, 120.0)	106.0 (90.0, 121.0)	0.02
Missing data	0 (0)	0 (0)	
HDL-C, mg/dL	64.0 (54.0, 75.0)	64.0 (55.0, 75.0)	0.01
Missing data	0 (0)	0 (0)	
Non-HDL-C, mg/dL	125.0 (108.0, 142.0)	125.0 (108.0, 141.0)	-0.004
Missing data	0 (0)	0 (0)	
Blood glucose, mg/dL	93.0 (88.0, 100.0)	93.0 (87.0, 99.0)	-0.06
Missing data	2 (0.01)	7 (0.043)	
HbA1c, %	5.1 (4.9, 5.5)	5.1 (4.9, 5.4)	-0.07
Missing data	4 (0.03)	0 (0)	
Smoking status, n (%)			
Never smoker	7,658 (50.0)	8,091 (50.1)	0.04
Former smoker	2,359 (15.3)	2,853 (17.7)	
Current smoker	5,277 (34.5)	5,177 (32.1)	
Missing data	26 (0.2)	28 (0.2)	
Drinking status, n (%)			
None	4,851 (31.7)	4,345 (26.9)	0.06
1-2 days/week	2,208 (14.4)	2,474 (15.3)	
3-6 days/week	3,027 (19.8)	3,599 (22.3)	
7 days/week	5,198 (33.9)	5,703 (35.3)	
Missing data	27 (0.2)	28 (0.2)	
Skipping breakfast, n (%)			
No	14,209 (92.8)	15,261 (94.5)	0.04
Yes	1,075 (7.0)	861 (5.3)	
Missing data	27 (0.2)	27 (0.2)	
Exercise habit, n (%)			

No	10,693 (69.8)	10,639 (65.9)	0.04
Yes	4,592 (30.0)	5,483 (34.0)	
Missing data	26 (0.2)	27 (0.2)	
Hypertension, n (%)	3,224 (21.1)	2,911 (18.0)	0.04
Diabetes, n (%)	823 (5.4)	661 (4.1)	0.03
Grip strength, kg	37.0 (28.0, 44.0)	38.0 (29.0, 45.0)	0.08
Missing data, n (%)	7,849 (51.3)	0 (0.0)	
Relative grip strength, kg/(kg/m^2)	1.7 (1.3, 2.0)	1.7 (1.3, 2.0)	0.05
Vertical jump, cm/kg	39.0 (33.0, 45.0)	40.0 (33.0, 46.0)	0.08
Missing data, n (%)	8,612 (56.2)	2,268 (14.0)	
Single-leg balance, sec.	30.0 (14.0, 63.0)	33.5 (15.0, 67.0)	0.05
Missing data, n (%)	8,394 (54.8)	1,805 (11.2)	
Forward bend, cm	8.0 (2.0, 13.0)	9.0 (3.0, 14.0)	0.08
Missing data, n (%)	8,420 (55.0)	1,842 (11.2)	
Reaction time, msec.	361.0 (331.0, 398.0)	352.0 (324.0, 386.0)	-0.18
Missing data, n (%)	8,416 (55.0)	1,834 (11.4)	
Dyslipidemia incidence, n (%)	3,970 (25.9)	6,919 (42.8)	0.18

BMI, body mass index; DBP, diastolic blood pressure; HbA1c, hemoglobin A1c; HDL-C, high-density lipoprotein cholesterol; LDL-C, low-density lipoprotein cholesterol; SBP, systolic blood pressure; TC, total cholesterol; TG, triglyceride.

Data are expressed as median (interquartile range) for continuous variable and number (percentage) for categorical variable.

[a] Values are expressed as Cohen's *d* for continuous variable and Cramer's *V* for categorical variable (30, 31).

Cohen's *d*: very small (0.01), small (0.20), medium (0.50), and large (0.80).

Cramer's *V*: negligible (<0.10), weak (<0.20), and moderate (<0.40).

References

30. Sawilowsky SS. New effect size rules of thumb. Journal of Modern Applied Statistical Methods. 2009;8(2):26.
31. Rea LM, Parker RA. Designing and conducting survey research: A comprehensive guide. San Francisco, CA: John Wiley & Sons; 2014.

eTable 2. Hazard ratios of the incidence of dyslipidemia according to septiles of relative grip strength in a sub-group analysis by age among men and women

	Relative grip strength, kg(kgm^2)[a]	Person-year	Case (%)	Age-adjusted HR (95% CI)	Model 1, HR (95% CI)[b]	Model 2, HR (95% CI)[c]
Men (n=9,941)						
Age <50 years						
S1 (n=613)	1.65 (1.54, 1.71)	1,740	336 (54.8)	1 (Reference)	1 (Reference)	1 (Reference)
S2 (n=615)	1.83 (1.80, 1.87)	1,847	348 (56.6)	0.997 (0.86, 1.16)	1.004 (0.86, 1.17)	1.01 (0.87, 1.17)
S3 (n=617)	1.96 (1.93, 2.00)	1,997	317 (51.4)	0.85 (0.73, 0.99)	0.86 (0.74, 1.001)	0.86 (0.73, 1.001)
S4 (n=613)	2.08 (2.05, 2.11)	2,032	316 (51.5)	0.84 (0.72, 0.98)	0.84 (0.72, 0.98)	0.84 (0.72, 0.98)
S5 (n=616)	2.20 (2.17, 2.24)	2,143	280 (45.5)	0.71 (0.61, 0.83)	0.72 (0.61, 0.84)	0.72 (0.61, 0.85)
S6 (n=615)	2.34 (2.29, 2.39)	2,175	277 (45.0)	0.69 (0.59, 0.81)	0.70 (0.59, 0.82)	0.70 (0.59, 0.82)
S7 (n=614)	2.58 (2.50, 2.70)	2,314	229 (37.3)	0.54 (0.46, 0.64)	0.54 (0.46, 0.64)	0.54 (0.45, 0.64)
P for trend				<0.001	<0.001	<0.001
Age ≥50 years						
S1 (n=803)	1.40 (1.29, 1.52)	2,160	384 (47.8)	1 (Reference)	1 (Reference)	1 (Reference)
S2 (n=808)	1.64 (1.50, 1.71)	2,290	334 (41.3)	0.83 (0.71, 0.96)	0.83 (0.72, 0.96)	0.82 (0.70, 0.94)
S3 (n=805)	1.75 (1.61, 1.84)	2,256	358 (44.5)	0.89 (0.77, 1.03)	0.90 (0.78, 1.004)	0.87 (0.75, 1.01)
S4 (n=806)	1.86 (1.72, 1.94)	2,351	341 (42.3)	0.82 (0.71, 0.95)	0.83 (0.72, 0.96)	0.80 (0.69, 0.93)
S5 (n=807)	1.97 (1.82, 2.05)	2,413	339 (42.0)	0.80 (0.69, 0.92)	0.81 (0.70, 0.94)	0.78 (0.67, 0.90)
S6 (n=806)	2.10 (1.96, 2.18)	2,452	320 (39.7)	0.75 (0.64, 0.87)	0.75 (0.65, 0.88)	0.73 (0.62, 0.85)
S7 (n=803)	2.33 (2.19, 2.44)	2,615	279 (34.7)	0.61 (0.53, 0.72)	0.63 (0.54, 0.73)	0.59 (0.50, 0.69)
P for trend				<0.001	<0.001	<0.001
Women (n=6,208)						
Age <50 years						
S1 (n=489)	1.01 (0.93, 1.06)	1,775	200 (40.9)	1 (Reference)	1 (Reference)	1 (Reference)
S2 (n=491)	1.16 (1.13, 1.20)	1,903	177 (36.0)	0.84 (0.68, 1.03)	0.87 (0.71, 1.06)	0.90 (0.73, 1.10)
S3 (n=491)	1.26 (1.23, 1.29)	1,869	181 (36.9)	0.86 (0.70, 1.05)	0.89 (0.72, 1.09)	0.91 (0.74, 1.12)
S4 (n=490)	1.35 (1.32, 1.38)	1,964	184 (37.6)	0.82 (0.67, 1.00)	0.84 (0.69, 1.03)	0.86 (0.70, 1.06)

S5 (n=491)	1.43 (1.40, 1.47)	1,930	171 (34.8)	0.80 (0.65, 0.98)	0.83 (0.67, 1.02)	0.86 (0.70, 1.07)
S6 (n=491)	1.54 (1.50, 1.58)	1,949	145 (29.5)	0.67 (0.54, 0.83)	0.69 (0.56, 0.86)	0.72 (0.57, 0.90)
S7 (n=489)	1.70 (1.65, 1.79)	1,988	120 (24.5)	0.54 (0.43, 0.68)	0.57 (0.45, 0.72)	0.60 (0.47, 0.76)
P for trend				<0.001	<0.001	<0.001
Age ≥50 years						
S1 (n=395)	0.86 (0.79, 0.93)	1,064	193 (48.9)	1 (Reference)	1 (Reference)	1 (Reference)
S2 (n=397)	1.01 (0.96, 1.04)	1,082	197 (49.6)	0.996 (0.82, 1.22)	1.02 (0.83, 1.24)	1.01 (0.83, 1.23)
S3 (n=398)	1.10 (1.04, 1.13)	1,109	175 (44.0)	0.87 (0.71, 1.07)	0.88 (0.72, 1.08)	0.87 (0.71, 1.07)
S4 (n=396)	1.18 (1.12, 1.21)	1,112	173 (43.7)	0.85 (0.70, 1.05)	0.88 (0.71, 1.08)	0.87 (0.70, 1.07)
S5 (n=398)	1.26 (1.19, 1.30)	1,095	186 (46.7)	0.94 (0.77, 1.15)	0.95 (0.77, 1.16)	0.94 (0.76, 1.16)
S6 (n=397)	1.35 (1.29, 1.39)	1,169	200 (50.4)	0.96 (0.78, 1.17)	0.995 (0.82, 1.21)	0.98 (0.80, 1.21)
S7 (n=395)	1.51 (1.45, 1.60)	1,170	159 (40.3)	0.76 (0.61, 0.93)	0.77 (0.63, 0.96)	0.77 (0.61, 0.96)
P for trend				0.036	0.07	0.10

CI, confidence interval; HR, hazard ratio.

[a] Data are expressed as median (interquartile range).

[b] Adjusted for age (continuous variable), smoking status (never smoker, former smoker, or current smoker), drinking status (none, 1-3 days/week, 4-6 days/week, or 7 days/week), breakfast skipping (no or yes), diabetes (no or yes), and hypertension (no or yes).

[c] Additionally adjusted for vertical jump (continuous variable), single-leg balance (continuous variable), forward bend (continuous variable), and reaction time (continuous variable).

eTable 3. Hazard ratios of the incidence of dyslipidemia according to septiles of relative grip strength excluding those who developed dyslipidemia within 2-year after the onset of follow-up among men and women

	Relative grip strength, kg(kg/m^2)[a]	Person-year	Case (%)	Age-adjusted HR (95% CI)	Model 1, HR (95% CI)[b]	Model 2, HR (95% CI)[c]
Men (n=7,150)						
Dyslipidemia						
S1 (n=930)	1.51 (1.36, 1.63)	3,484	234 (25.2)	1 (Reference)	1 (Reference)	1 (Reference)
S2 (n=972)	1.74 (1.60, 1.82)	3,757	231 (23.8)	0.91 (0.76, 1.09)	0.91 (0.76, 1.09)	0.91 (0.75, 1.09)
S3 (n=997)	1.87 (1.72, 1.95)	3,871	250 (25.1)	0.94 (0.78, 1.12)	0.94 (0.79, 1.12)	0.93 (0.78, 1.12)
S4 (n=1,005)	1.97 (1.84, 2.07)	4,009	243 (24.2)	0.87 (0.72, 1.04)	0.86 (0.72, 1.03)	0.86 (0.71, 1.03)
S5 (n=1,055)	2.09 (1.95, 2.19)	4,232	251 (23.8)	0.86 (0.72, 1.02)	0.86 (0.72, 1.02)	0.85 (0.71, 1.02)
S6 (n=1,061)	2.23 (2.08, 2.33)	4,297	237 (22.3)	0.78 (0.65, 0.94)	0.78 (0.65, 0.94)	0.78 (0.64, 0.94)
S7 (n=1,130)	2.47 (2.31, 2.61)	4,648	221 (19.6)	0.67 (0.56, 0.80)	0.66 (0.55, 0.80)	0.65 (0.54, 0.79)
P for trend				<0.001	<0.001	<0.001
Women (n=4,898)						
Dyslipidemia						
S1 (n=676)	0.94 (0.85, 1.03)	2,645	185 (27.4)	1 (Reference)	1 (Reference)	1 (Reference)
S2 (n=694)	1.12 (1.02, 1.17)	2,803	180 (25.9)	0.94 (0.77, 1.15)	0.96 (0.78, 1.18)	0.96 (0.78, 1.18)
S3 (n=693)	1.22 (1.11, 1.27)	2,780	160 (23.1)	0.82 (0.66, 1.01)	0.84 (0.68, 1.03)	0.84 (0.67, 1.04)
S4 (n=694)	1.30 (1.19, 1.35)	2,883	165 (23.8)	0.79 (0.64, 0.98)	0.81 (0.66, 1.004)	0.81 (0.65, 1.01)
S5 (n=689)	1.38 (1.28, 1.45)	2,822	157 (22.8)	0.80 (0.65, 0.99)	0.82 (0.66, 1.02)	0.82 (0.66, 1.03)
S6 (n=709)	1.49 (1.37, 1.55)	2,960	166 (23.4)	0.80 (0.65, 0.98)	0.83 (0.67, 1.03)	0.83 (0.67, 1.03)
S7 (n=743)	1.65 (1.55, 1.74)	3,020	138 (18.6)	0.64 (0.51, 0.80)	0.66 (0.53, 0.83)	0.66 (0.52, 0.84)
P for trend				<0.001	<0.001	0.001

CI, confidence interval; HR, hazard ratio.

[a] Data are expressed as median (interquartile range).

[b] Adjusted for age (continuous variable), smoking status (never smoker, former smoker, or current smoker), drinking status (none, 1-3 days/week, 4-6 days/week, or 7 days/week), breakfast skipping (no or yes), diabetes (no or yes), and hypertension (no or yes).

[c] Additionally adjusted for vertical jump (continuous variable), single-leg balance (continuous variable), forward bend (continuous variable), and reaction time (continuous

variable).

eTable 4. Hazard ratios of the incidence of dyslipidemia according to septiles of relative grip strength using a more conservative criteria for dyslipidemia among men and women

	Relative grip strength, kg(kg/m^2)[a]	Person-year	Case (%)	Age-adjusted HR (95% CI)	Model 1, HR (95% CI)[b]	Model 2, HR (95% CI)[c]
Men (n=9,941)						
Dyslipidemia						
S1 (n=1,416)	1.51 (1.36, 1.63)	4,295	464 (32.8)	1 (Reference)	1 (Reference)	1 (Reference)
S2 (n=1,423)	1.75 (1.61, 1.82)	4,552	401 (28.2)	0.83 (0.72, 0.95)	0.83 (0.73, 0.95)	0.82 (0.72, 0.94)
S3 (n=1,422)	1.87 (1.73, 1.95)	4,644	411 (28.9)	0.84 (0.73, 0.95)	0.85 (0.74, 0.97)	0.83 (0.73, 0.95)
S4 (n=1,419)	1.97 (1.84, 2.07)	4,805	401 (28.3)	0.80 (0.70, 0.91)	0.81 (0.71, 0.92)	0.79 (0.69, 0.90)
S5 (n=1,423)	2.09 (1.95, 2.19)	4,887	388 (27.3)	0.76 (0.67, 0.87)	0.78 (0.68, 0.89)	0.75 (0.66, 0.86)
S6 (n=1,421)	2.23 (2.08, 2.33)	5,020	344 (24.2)	0.66 (0.57, 0.76)	0.67 (0.58, 0.77)	0.65 (0.56, 0.75)
S7 (n=1,417)	2.46 (2.30, 2.60)	5,260	284 (20.0)	0.53 (0.46, 0.61)	0.54 (0.46, 0.62)	0.51 (0.44, 0.60)
P for trend				<0.001	<0.001	<0.001
Women (n=6,208)						
Dyslipidemia						
S1 (n=884)	0.94 (0.85, 1.02)	2,987	261 (29.5)	1 (Reference)	1 (Reference)	1 (Reference)
S2 (n=888)	1.09 (1.02, 1.16)	3,160	230 (25.9)	0.84 (0.70, 0.996)	0.85 (0.72, 1.02)	0.86 (0.72, 1.03)
S3 (n=889)	1.20 (1.10, 1.26)	3,101	238 (26.8)	0.88 (0.74, 1.05)	0.90 (0.76, 1.08)	0.91 (0.76, 1.09)
S4 (n=886)	1.29 (1.18, 1.35)	3,206	228 (25.7)	0.83 (0.70, 0.991)	0.85 (0.71, 1.02)	0.85 (0.71, 1.03)
S5 (n=889)	1.36 (1.27, 1.44)	3,156	247 (27.8)	0.92 (0.77, 1.10)	0.94 (0.79, 1.12)	0.95 (0.79, 1.14)
S6 (n=888)	1.47 (1.36, 1.54)	3,283	204 (23.0)	0.74 (0.61, 0.88)	0.77 (0.64, 0.92)	0.77 (0.64, 0.93)
S7 (n=884)	1.64 (1.53, 1.74)	3,271	180 (20.4)	0.65 (0.54, 0.79)	0.68 (0.56, 0.82)	0.69 (0.56, 0.85)
P for trend				<0.001	<0.001	0.001

CI, confidence interval; HR, hazard ratio.

Participants were determined to have dyslipidemia when an individual met the criteria for dyslipidemia at least twice during the follow-up period.

[a] Data are expressed as median (interquartile range).

[b] Adjusted for age (continuous variable), smoking status (never smoker, former smoker, or current smoker), drinking status (none, 1-3 days/week, 4-6 days/week, or 7 days/week), breakfast skipping (no or yes), diabetes (no or yes), and hypertension (no or yes).

[c] Additionally adjusted for vertical jump (continuous variable), single-leg balance (continuous variable), forward bend (continuous variable), and reaction time (continuous variable).

eTable 5. Hazard ratios of the incidence of dyslipidemia according to septiles of absolute grip strength among men and women

	Grip strength, kg[a]	Person-year	Case (%)	Model 2[b]	Model 3 (+ BMI)[c]	+ Body weight[c]	+ Height[c]	+ Height and BMI[c]
Men (n=9,941)								
Dyslipidemia								
S1 (n=1,546)	35.0 (32.0, 37.0)	4,697	633 (40.9)	1 (Reference)	1 (Reference)	1 (Reference)	1 (Reference)	1 (Reference)
S2 (n=1,621)	40.0 (38.0, 42.0)	4,947	767 (47.3)	1.14 (1.03, 1.27)	1.08 (0.97, 1.20)	1.05 (0.95, 1.17)	1.16 (1.04, 1.29)	1.08 (0.97, 1.20)
S3 (n=1,435)	43.0 (39.0, 44.0)	4,452	623 (43.4)	1.06 (0.95, 1.18)	0.98 (0.87, 1.09)	0.94 (0.84, 1.05)	1.08 (0.96, 1.21)	0.98 (0.87, 1.10)
S4 (n=1,347)	45.0 (41.0, 46.0)	4,148	598 (44.4)	1.08 (0.97, 1.21)	0.97 (0.87, 1.09)	0.93 (0.83, 1.05)	1.11 (0.99, 1.24)	0.98 (0.87, 1.10)
S5 (n=1,442)	47.0 (44.0, 49.0)	4,522	650 (45.1)	1.09 (0.97, 1.22)	0.96 (0.86, 1.08)	0.90 (0.81, 1.01)	1.12 (1.001, 1.26)	0.96 (0.86, 1.08)
S6 (n=1,285)	49.0 (47.0, 51.0)	3,997	596 (46.4)	1.12 (0.995, 1.25)	0.94 (0.84, 1.06)	0.88 (0.78, 0.99)	1.16 (1.03, 1.30)	0.94 (0.84, 1.06)
S7 (n=1,265)	54.0 (51.0, 56.0)	4,022	591 (46.7)	1.12 (0.997, 1.26)	0.90 (0.80, 1.02)	0.82 (0.72, 0.92)	1.17 (1.04, 1.33)	0.91 (0.80, 1.03)
P for trend				0.18	0.006	<0.001	0.04	0.011
Women (n=6,208)								
Dyslipidemia								
S1 (n=1,103)	22.0 (20.0, 23.0)	3,752	424 (38.4)	1 (Reference)	1 (Reference)	1 (Reference)	1 (Reference)	1 (Reference)
S2 (n=986)	25.0 (23.0, 26.0)	3,425	386 (39.1)	1.02 (0.88, 1.17)	1.01 (0.87, 1.15)	0.98 (0.85, 1.12)	1.03 (0.89, 1.18)	1.01 (0.88, 1.16)
S3 (n=820)	26.0 (25.0, 27.0)	2,678	318 (38.8)	1.02 (0.88, 1.19)	0.98 (0.85, 1.14)	0.95 (0.82, 1.10)	1.04 (0.90, 1.21)	0.98 (0.85, 1.14)
S4 (n=1,008)	28.0 (27.0, 29.0)	3,577	381 (37.8)	0.997 (0.87, 1.15)	0.95 (0.82, 1.09)	0.91 (0.79, 1.05)	1.02 (0.88, 1.18)	0.95 (0.82, 1.10)
S5 (n=874)	30.0 (28.0, 31.0)	3,079	355 (40.6)	1.15 (0.997, 1.33)	1.06 (0.92, 1.23)	1.004 (0.87, 1.16)	1.19 (1.02, 1.38)	1.07 (0.92, 1.24)
S6 (n=732)	32.0 (29.0, 33.0)	2,414	323 (44.1)	1.21 (1.04, 1.40)	1.13 (0.97, 1.31)	1.04 (0.90, 1.22)	1.26 (1.07, 1.46)	1.13 (0.97, 1.32)
S7 (n=685)	35.0 (33.0, 36.0)	2,254	274 (40.0)	1.19 (1.01, 1.39)	1.02 (0.87, 1.20)	0.93 (0.78, 1.09)	1.25 (1.05, 1.47)	1.03 (0.87, 1.22)
P for trend				0.002	0.24	0.91	<0.001	0.22

BMI, body mass index.

[a] Data are expressed as median (interquartile range).

[b] Hazard ratios and 95% confidence intervals were calculated after adjustment for age (continuous variable), smoking status (never smoker, former smoker, or current smoker), drinking status (none, 1-3 days/week, 4-6 days/week, or 7 days/week), breakfast skipping (no or yes), diabetes (no or yes), hypertension (no or yes), vertical jump (continuous variable), single-leg balance (continuous variable), forward bend (continuous variable), and reaction time (continuous variable).

[c] Additionally adjusted for the variables (body weight, height, BMI, and height + BMI) shown in the table, respectively.

eTable 6. Association between the septiles of each physical fitness and the incidence of dyslipidemia among men and women

	Median (interquartile range)	Person-year	Case (%)	Age-adjusted HR (95% CI)	Model 1, HR (95% CI)[a]	Model 2, HR (95% CI)[b]	Model 3, HR (95% CI)[c]
Men (n=9,941)							
Vertical jump, cm							
S1 (n=1,592)	36.0 (32.1, 39.6)	4,779	692 (43.5)	1 (Reference)	1 (Reference)	1 (Reference)	1 (Reference)
S2 (n=1,482)	40.9 (37.5, 44.0)	4,634	652 (44.0)	0.98 (0.87, 1.11)	0.98 (0.87, 1.10)	0.97 (0.86, 1.10)	1.01 (0.89, 1.13)
S3 (n=1,361)	42.7 (39.6, 46.7)	4,276	616 (45.2)	1.00 (0.89, 1.12)	0.999 (0.89, 1.12)	0.99 (0.88, 1.11)	1.05 (0.93, 1.18)
S4 (n=1,658)	44.7 (41.6, 48.1)	5,085	756 (45.6)	1.03 (0.92, 1.16)	1.04 (0.92, 1.16)	1.03 (0.91, 1.15)	1.08 (0.96, 1.21)
S5 (n=1,334)	46.5 (43.7, 50.0)	4,033	599 (44.9)	1.03 (0.91, 1.16)	1.04 (0.92, 1.17)	1.03 (0.91, 1.17)	1.10 (0.97, 1.25)
S6 (n=1,209)	49.2 (45.8, 53.0)	3,816	575 (47.5)	1.04 (0.93, 1.18)	1.06 (0.94, 1.19)	1.05 (0.93, 1.19)	1.13 (0.998, 1.28)
S7 (n=1,306)	54.0 (49.9, 58.0)	4,163	569 (43.6)	0.95 (0.84, 1.07)	0.97 (0.86, 1.09)	0.96 (0.85, 1.09)	1.07 (0.94, 1.21)
P for trend				0.969	0.680	0.718	0.047
Single-leg balance, sec							
S1 (n=1,580)	6.5 (5.0, 10.0)	4,723	718 (45.4)	1 (Reference)	1 (Reference)	1 (Reference)	1 (Reference)
S2 (n=1,354)	15.8 (11.0, 21.6)	4,067	634 (46.8)	1.03 (0.92, 1.15)	1.03 (0.92, 1.15)	1.03 (0.92, 1.15)	1.04 (0.93, 1.16)
S3 (n=1,407)	23.1 (16.7, 34.4)	4,208	630 (44.8)	0.98 (0.88, 1.10)	0.997 (0.89, 1.11)	0.99 (0.88, 1.10)	0.99 (0.89, 1.11)
S4 (n=1,399)	34.4 (24.4, 49.7)	4,397	605 (43.2)	0.91 (0.81, 1.02)	0.92 (0.82, 1.04)	0.92 (0.82, 1.03)	0.92 (0.82, 1.04)
S5 (n=1,392)	49.9 (35.3, 67.3)	4,211	668 (48.0)	1.04 (0.93, 1.16)	1.06 (0.95, 1.18)	1.05 (0.94, 1.17)	1.06 (0.95, 1.19)
S6 (n=1,406)	69.8 (52.7, 93.7)	4,622	620 (44.1)	0.89 (0.80, 1.000)	0.91 (0.81, 1.02)	0.89 (0.79, 0.997)	0.89 (0.80, 1.003)
S7 (n=1,403)	120.6 (87.2, 166.4)	4,557	584 (41.6)	0.85 (0.76, 0.96)	0.88 (0.78, 0.99)	0.86 (0.76, 0.97)	0.91 (0.80, 1.03)
P for trend				0.002	0.010	0.004	0.031
Trunk flexibility, cm							
S1 (n=1,518)	-6.0 (-9.3, -4.0)	4,507	712 (46.9)	1 (Reference)	1 (Reference)	1 (Reference)	1 (Reference)
S2 (n=1,479)	0.5 (-0.9, 1.1)	4,508	657 (44.5)	0.93 (0.83, 1.04)	0.93 (0.83, 1.04)	0.92 (0.82, 1.04)	0.94 (0.83, 1.05)
S3 (n=1,518)	4.0 (3.0, 5.0)	4,640	685 (45.1)	0.93 (0.84, 1.04)	0.94 (0.84, 1.05)	0.93 (0.83, 1.04)	0.94 (0.84, 1.05)
S4 (n=1,465)	7.0 (6.0, 7.9)	4,477	665 (45.4)	0.94 (0.84, 1.06)	0.95 (0.85, 1.07)	0.94 (0.84, 1.06)	0.96 (0.85, 1.08)
S5 (n=1,309)	9.7 (9.0, 10.0)	4,050	590 (45.0)	0.92 (0.82, 1.04)	0.93 (0.83, 1.05)	0.92 (0.82, 1.03)	0.93 (0.83, 1.05)

S6 (n=1,334)	13.0 (12.0, 14.0)	4,235	601 (45.0)	0.91 (0.81, 1.02)	0.93 (0.83, 1.05)	0.93 (0.83, 1.04)	0.96 (0.85, 1.07)
S7 (n=1,319)	17.3 (16.0, 20.0)	4,368	548 (41.6)	0.81 (0.72, 0.91)	0.82 (0.73, 0.93)	0.81 (0.72, 0.91)	0.85 (0.75, 0.96)
P for trend				0.002	0.009	0.005	0.044
Whole body reaction time, sec							
S1 (n=1,456)	292.2 (281.4, 299.4)	4,710	665 (45.7)	1 (Reference)	1 (Reference)	1 (Reference)	1 (Reference)
S2 (n=1,426)	313.1 (307.9, 321.2)	4,412	657 (46.0)	1.05 (0.94, 1.17)	1.05 (0.94, 1.18)	1.05 (0.94, 1.17)	1.03 (0.92, 1.15)
S3 (n=1,422)	328.8 (322.0, 337.7)	4,366	639 (44.9)	1.03 (0.92, 1.15)	1.02 (0.91, 1.14)	1.01 (0.90, 1.13)	0.995 (0.89, 1.11)
S4 (n=1,416)	342.4 (334.8, 352.9)	4,373	664 (46.9)	1.07 (0.96, 1.19)	1.06 (0.95, 1.19)	1.04 (0.93, 1.16)	1.03 (0.93, 1.15)
S5 (n=1,410)	358.0 (348.3, 371.7)	4,359	624 (44.3)	1.000 (0.89, 1.12)	0.99 (0.88, 1.11)	0.97 (0.86, 1.09)	0.95 (0.85, 1.07)
S6 (n=1,415)	379.0 (367.8, 395.9)	4,396	610 (43.1)	0.98 (0.87, 1.10)	0.96 (0.86, 1.08)	0.95 (0.84, 1.06)	0.93 (0.83, 1.05)
S7 (n=1,396)	423.5 (402.2, 453.8)	4,169	600 (43.0)	1.01 (0.90, 1.13)	0.98 (0.88, 1.10)	0.96 (0.85, 1.08)	0.94 (0.84, 1.05)
P for trend				0.521	0.252	0.117	0.056
Women (n=6,208)							
Vertical jump, cm[d]							
S1 (n=996)	26.0 (23.0, 28.9)	3,247	413 (41.5)	1 (Reference)	1 (Reference)	1 (Reference)	1 (Reference)
S2 (n=1,000)	29.5 (27.0, 31.4)	3,414	412 (41.2)	0.93 (0.80, 1.09)	0.95 (0.81, 1.11)	0.93 (0.79, 1.09)	0.992 (0.85, 1.16)
S3 (n=897)	32.4 (29.8, 34.0)	3,161	345 (38.4)	0.90 (0.76, 1.07)	0.92 (0.78, 1.10)	0.90 (0.76, 1.08)	0.98 (0.82, 1.17)
S4 (n=865)	32.0 (30.0, 34.5)	2,752	371 (42.9)	0.993 (0.85, 1.16)	1.02 (0.87, 1.19)	0.992 (0.85, 1.16)	1.09 (0.93, 1.28)
S5 (n=777)	35.1 (32.5, 37.1)	2,721	291 (37.4)	0.86 (0.73, 1.03)	0.89 (0.75, 1.05)	0.86 (0.72, 1.02)	0.97 (0.81, 1.16)
S6 (n=931)	37.0 (34.0, 39.0)	3,246	356 (38.2)	0.90 (0.77, 1.05)	0.93 (0.80, 1.09)	0.90 (0.76, 1.05)	1.04 (0.88, 1.22)
S7 (n=742)	40.8 (37.3, 43.1)	2,637	274 (36.9)	0.84 (0.70, 1.01)	0.87 (0.72, 1.04)	0.82 (0.68, 0.997)	0.98 (0.80, 1.19)
P for trend				0.061	0.157	0.060	0.887
Single-leg balance, sec							
S1 (n=943)	7.9 (5.0, 12.0)	2,975	394 (41.8)	1 (Reference)	1 (Reference)	1 (Reference)	1 (Reference)
S2 (n=874)	20.0 (14.5, 24.7)	2,952	348 (39.8)	0.91 (0.78, 1.06)	0.92 (0.79, 1.07)	0.91 (0.78, 1.07)	0.94 (0.80, 1.10)
S3 (n=878)	33.3 (24.6, 39.9)	3,003	358 (40.8)	0.92 (0.80, 1.07)	0.94 (0.81, 1.09)	0.93 (0.80, 1.08)	0.95 (0.82, 1.11)

S4 (n=881)	48.0 (36.8, 56.7)	3,002	378 (42.9)	0.98 (0.84, 1.13)	0.994 (0.86, 1.16)	0.98 (0.84, 1.14)	1.01 (0.87, 1.18)
S5 (n=875)	66.2 (51.9, 75.2)	3,063	337 (38.5)	0.85 (0.73, 1.001)	0.86 (0.73, 1.01)	0.85 (0.73, 1.01)	0.88 (0.75, 1.04)
S6 (n=883)	91.4 (73.0, 105.0)	3,035	347 (39.3)	0.90 (0.76, 1.05)	0.92 (0.78, 1.08)	0.91 (0.77, 1.07)	0.96 (0.81, 1.13)
S7 (n=875)	147.1 (120.9, 206.6)	3,151	299 (34.2)	0.74 (0.63, 0.87)	0.75 (0.64, 0.89)	0.75 (0.63, 0.88)	0.81 (0.68, 0.96)
P for trend				0.001	0.002	0.002	0.039
Trunk flexibility, cm							
S1 (n=941)	1.0 (-2.0, 3.1)	3,174	363 (38.6)	1 (Reference)	1 (Reference)	1 (Reference)	1 (Reference)
S2 (n=928)	7.0 (6.0, 8.0)	3,168	374 (40.3)	1.04 (0.88, 1.22)	1.04 (0.89, 1.23)	1.04 (0.88, 1.22)	1.04 (0.89, 1.23)
S3 (n=904)	10.0 (9.0, 11.0)	2,961	376 (41.6)	1.11 (0.94, 1.31)	1.11 (0.94, 1.31)	1.11 (0.94, 1.31)	1.09 (0.92, 1.29)
S4 (n=899)	12.1 (11.3, 13.0)	3,090	356 (39.6)	1.01 (0.86, 1.18)	1.01 (0.86, 1.19)	1.01 (0.86, 1.19)	1.02 (0.86, 1.19)
S5 (n=873)	14.9 (14.0, 15.4)	2,949	341 (39.0)	1.01 (0.86, 1.18)	1.03 (0.88, 1.20)	1.01 (0.86, 1.19)	1.02 (0.87, 1.19)
S6 (n=852)	17.1 (16.2, 18.0)	3,011	332 (38.9)	0.96 (0.82, 1.14)	0.98 (0.83, 1.15)	0.97 (0.82, 1.15)	0.97 (0.82, 1.14)
S7 (n=811)	21.0 (19.9, 23.0)	2,826	320 (39.4)	0.997 (0.83, 1.19)	1.02 (0.85, 1.23)	1.02 (0.84, 1.22)	1.03 (0.85, 1.24)
P for trend				0.439	0.638	0.581	0.644
Whole body reaction time, sec							
S1 (n=896)	310.5 (298.7, 318.8)	3,155	347 (38.7)	1 (Reference)	1 (Reference)	1 (Reference)	1 (Reference)
S2 (n=902)	335.1 (329.6, 342.1)	3,076	382 (42.3)	1.16 (0.994, 1.36)	1.16 (0.992, 1.36)	1.15 (0.98, 1.35)	1.12 (0.96, 1.31)
S3 (n=890)	352.6 (346.4, 360.7)	3,019	361 (40.6)	1.10 (0.93, 1.30)	1.10 (0.93, 1.30)	1.08 (0.91, 1.28)	1.05 (0.88, 1.24)
S4 (n=877)	367.9 (361.4, 377.2)	3,066	346 (39.4)	1.04 (0.88, 1.22)	1.04 (0.88, 1.22)	1.01 (0.86, 1.19)	0.97 (0.82, 1.14)
S5 (n=882)	386.1 (379.5, 397.2)	2,978	349 (39.6)	1.07 (0.91, 1.26)	1.06 (0.90, 1.24)	1.03 (0.87, 1.21)	0.99 (0.84, 1.17)
S6 (n=888)	410.4 (401.6, 422.2)	3,036	341 (38.4)	1.04 (0.88, 1.23)	1.03 (0.87, 1.21)	0.99 (0.83, 1.17)	0.93 (0.78, 1.10)
S7 (n=874)	457.8 (437.1, 487.8)	2,850	335 (38.3)	1.07 (0.91, 1.26)	1.05 (0.89, 1.24)	1.001 (0.84, 1.19)	0.95 (0.80, 1.13)
P for trend				0.861	0.647	0.262	0.063

CI, confidence interval; HR, hazard ratio.

[a] Adjusted for age (continuous variable), smoking status (never smoker, former smoker, or current smoker), drinking status (none, 1-3 days/week, 4-6 days/week, or 7 days/week), breakfast skipping (no or yes), diabetes (no or yes), and hypertension (no or yes).

[b] Additionally adjusted for the mutual physical fitness including grip strength (continuous variable), vertical jump (continuous variable), single-leg balance (continuous variable), forward bend (continuous variable), and reaction time (continuous variable).

[c] Additionally adjusted for BMI (continuous variable).

[d] The numbers of participants and cases may not sum to the numbers shown in the text due to multiple imputation.

eTable 7. Hazard ratios of the incidence of dyslipidemia according to septiles of relative vertical jump and trunk flexibility in complete-cases analysis

	Median (interquartile range)	Person-year	Case (%)	Model 1, HR (95% CI)[a]	Model 2, HR (95% CI)[b]	Model 3, HR (95% CI)[c]
Men						
Relative vertical jump, cm/(kg/m^2)						
S1 (n=1,253)	1.50 (1.29, 1.65)	3,556	606 (48.4)	1 (Reference)	1 (Reference)	
S2 (n=1,255)	1.74 (1.60, 1.89)	3,603	635 (50.6)	1.05 (0.93, 1.17)	1.05 (0.93, 1.17)	
S3 (n=1,254)	1.86 (1.73, 2.04)	3,774	611 (48.7)	0.97 (0.87, 1.09)	0.97 (0.87, 1.09)	
S4 (n=1,258)	1.97 (1.84, 2.18)	3,866	569 (45.2)	0.89 (0.79, 1.00)	0.89 (0.80, 1.004)	
S5 (n=1,254)	2.08 (1.95, 2.32)	4,060	539 (43.0)	0.80 (0.71, 0.90)	0.80 (0.71, 0.91)	
S6 (n=1,256)	2.25 (2.10, 2.50)	4,038	538 (42.8)	0.80 (0.71, 0.90)	0.80 (0.71, 0.91)	
S7 (n=1,252)	2.56 (2.33, 2.84)	4,378	425 (33.9)	0.59 (0.52, 0.66)	0.56 (0.52, 0.67)	
P for trend				<0.001	<0.001	
Trunk flexibility, cm						
S1 (n=1,396)	-6.0 (-10.0, -4.0)	4,154	653 (46.8)	1 (Reference)	1 (Reference)	1 (Reference)
S2 (n=1,288)	1.0 (-1.0, 1.0)	3,970	564 (43.8)	0.93 (0.83, 1.05)	0.92 (0.82, 1.04)	0.94 (0.84, 1.05)
S3 (n=1,412)	3.0 (4.0, 5.0)	4,306	635 (45.0)	0.96 (0.86, 1.08)	0.95 (0.85, 1.07)	0.96 (0.86, 1.08)
S4 (n=1,367)	7.0 (6.0, 8.0)	4,167	610 (44.6)	0.97 (0.86, 1.08)	0.96 (0.85, 1.07)	0.97 (0.87, 1.08)
S5 (n=1,177)	10.0 (9.0, 10.0)	3,705	535 (45.5)	0.95 (0.84, 1.07)	0.94 (0.83, 1.06)	0.94 (0.84, 1.06)
S6 (n=1,172)	13.0 (12.0, 14.0)	3,737	525 (44.8)	0.94 (0.84, 1.06)	0.93 (0.83, 1.05)	0.96 (0.85, 1.08)
S7 (n=1,156)	18.0 (16.0, 20.0)	3,845	475 (41.1)	0.83 (0.74, 0.94)	0.82 (0.73, 0.93)	0.86 (0.76, 0.98)
P for trend				0.023	0.013	0.081
Women						
Relative vertical jump, cm/(kg/m^2)						
S1 (n=1,253)	1.08 (0.94, 1.22)	2,267	331 (45.8)	1 (Reference)	1 (Reference)	
S2 (n=1,255)	1.29 (1.17, 1.42)	2,477	301 (41.5)	0.82 (0.70, 0.96)	0.80 (0.68, 0.94)	
S3 (n=1,254)	1.42 (1.30, 1.55)	2,372	287 (39.6)	0.84 (0.71, 0.98)	0.83 (0.71, 0.98)	
S4 (n=1,258)	1.54 (1.40, 1.67)	2,498	302 (41.5)	0.84 (0.72, 0.98)	0.82 (0.70, 0.96)	

S5 (n=1,254)	1.64 (1.50, 1.79)	2,559	248 (34.3)	0.68 (0.57, 0.80)	0.66 (0.56, 0.78)
S6 (n=1,256)	1.77 (1.63, 1.91)	2,618	260 (35.8)	0.71 (0.60, 0.84)	0.69 (0.58, 0.81)
S7 (n=1,252)	2.02 (1.85, 2.20)	2,638	221 (30.6)	0.59 (0.50, 0.70)	0.57 (0.48, 0.68)
P for trend				<0.001	<0.001

CI, confidence interval; HR, hazard ratio.

[a] Adjusted for age (continuous variable), smoking status (never smoker, former smoker, or current smoker), drinking status (none, 1-3 days/week, 4-6 days/week, or 7 days/week), breakfast skipping (no or yes), diabetes (no or yes), and hypertension (no or yes).

[b] Additionally adjusted for the mutual physical fitness including grip strength (continuous variable), vertical jump (continuous variable), single-leg balance (continuous variable), forward bend (continuous variable), and reaction time (continuous variable).

[c] Additionally adjusted for BMI (continuous variable).

eTable 8. Hazard ratios of the incidence of dyslipidemia according to septiles of relative grip strength and vertical jump after adjusted for serum lipid profile at baseline

	Median (interquartile range)	Person-year	Case (%)	+ serum lipid profile, HR (95% CI)[a]
Men (n=9,941)				
Relative grip strength				
S1 (n=1,416)	1.51 (1.36, 1.63)	3,900	720 (50.8)	1 (Reference)
S2 (n=1,423)	1.75 (1.61, 1.82)	4,137	682 (47.9)	0.98 (0.88, 1.08)
S3 (n=1,422)	1.87 (1.73, 1.95)	4,253	675 (47.5)	0.97 (0.87, 1.08)
S4 (n=1,419)	1.97 (1.84, 2.07)	4,383	657 (46.3)	0.99 (0.89, 1.10)
S5 (n=1,423)	2.09 (1.95, 2.19)	4,556	619 (43.5)	0.94 (0.84, 1.05)
S6 (n=1,421)	2.23 (2.08, 2.33)	4,627	597 (42.0)	0.95 (0.85, 1.06)
S7 (n=1,417)	2.46 (2.30, 2.60)	4,829	508 (35.9)	0.87 (0.78, 0.99)
P for trend				0.038
Relative vertical jump				
S1 (n=1,419)	1.50 (1.28, 1.64)	3,990	691 (48.7)	1 (Reference)
S2 (n=1,421)	1.73 (1.60, 1.89)	4,096	716 (50.4)	1.02 (0.91, 1.14)
S3 (n=1,421)	1.86 (1.73, 2.03)	4,292	693 (48.7)	1.04 (0.92, 1.17)
S4 (n=1,420)	1.97 (1.84, 2.18)	4,351	647 (45.5)	0.96 (0.84, 1.09)
S5 (n=1,422)	2.09 (1.96, 2.31)	4,573	614 (43.2)	0.90 (0.78, 1.03)
S6 (n=1,422)	2.25 (2.10, 2.48)	4,569	603 (42.7)	0.95 (0.82, 1.09)
S7 (n=1,417)	2.56 (2.34, 2.84)	4,915	491 (34.7)	0.80 (0.68, 0.94)
P for trend				0.006
Women (n=6,208)				
Relative grip strength				
S1 (n=884)	0.94 (0.85, 1.02)	2,839	393 (44.5)	1 (Reference)
S2 (n=888)	1.09 (1.02, 1.16)	2,985	374 (42.1)	1.05 (0.91, 1.21)
S3 (n=889)	1.20 (1.10, 1.26)	2,978	356 (40.0)	0.99 (0.85, 1.14)
S4 (n=886)	1.29 (1.18, 1.35)	3,076	357 (40.3)	1.14 (0.98, 1.32)
S5 (n=889)	1.37 (1.27, 1.44)	3,025	357 (40.2)	1.15 (0.99, 1.33)
S6 (n=888)	1.47 (1.36, 1.54)	3,118	345 (38.9)	1.08 (0.93, 1.26)
S7 (n=884)	1.64 (1.53, 1.74)	3,158	279 (31.6)	0.99 (0.84, 1.17)
P for trend				0.495
Relative vertical jump				
S1 (n=885)	1.06 (0.92, 1.20)	2,756	422 (47.7)	1 (Reference)
S2 (n=888)	1.28 (1.16, 1.41)	2,978	380 (42.8)	0.93 (0.80, 1.09)
S3 (n=888)	1.41 (1.29, 1.55)	2,910	366 (41.2)	0.95 (0.80, 1.12)
S4 (n=886)	1.53 (1.39, 1.67)	3,022	375 (42.3)	0.996 (0.83, 1.20)
S5 (n=889)	1.63 (1.50, 1.78)	3,107	314 (35.4)	0.88 (0.72, 1.07)
S6 (n=888)	1.77 (1.63, 1.91)	3,204	322 (36.3)	0.87 (0.71, 1.08)
S7 (n=885)	2.02 (1.85, 2.21)	3,205	282 (31.9)	0.83 (0.65, 1.06)

P for trend	0.147

CI, confidence interval; HR, hazard ratio.

[a] Adjusted for age (continuous variable), smoking status (never smoker, former smoker, or current smoker), drinking status (none, 1-3 days/week, 4-6 days/week, or 7 days/week), breakfast skipping (no or yes), diabetes (no or yes), hypertension (no or yes), vertical jump (continuous variable), single-leg balance (continuous variable), forward bend (continuous variable), reaction time (continuous variable), serum LDL-cholesterol (continuous variable), triglycerides (continuous variable), and LDL-cholesterol (continuous variable) at baseline.
